# DMT and harmala alkaloids: an exploratory study of oral Acacia based formulations in healthy volunteers

**DOI:** 10.3389/fpsyt.2025.1545915

**Published:** 2025-08-15

**Authors:** Yvonne A. Bonomo, Amanda F. Norman, Lisa Collins, Margaret Ross, Justin Dwyer, Daniel Perkins, Jerome Sarris

**Affiliations:** ^1^ Department of Addiction Medicine, St Vincent's Hospital Melbourne, Melbourne, VIC, Australia; ^2^ Department of Medicine, University of Melbourne, Melbourne, VIC, Australia; ^3^ Psychosocial Cancer Care, St Vincent’s Hospital Melbourne, Melbourne, VIC, Australia; ^4^ Psychae Institute, Melbourne, VIC, Australia; ^5^ School of Population and Global Health, University of Melbourne, Melbourne, VIC, Australia; ^6^ Swinburne University of Technology, Center for Mental Health and Brain Sciences, Melbourne, VIC, Australia; ^7^ NICM Health Research Institute, Western Sydney University, Westmead, NSW, Australia; ^8^ Florey Institute of Neuroscience and Mental Health, Melbourne University, Melbourne, VIC, Australia

**Keywords:** ayahuasca, psychedelics, plant medicine, mental health, depression, anxiety, ethnobotany

## Abstract

**Introduction:**

Ayahuasca is a psychedelic compound of N, N, Dimethyltryptamine (DMT) and harmala alkaloids used for spiritual and medicinal applications in traditional settings. A range of potential psychotherapeutic mechanisms have been proposed for ayahuasca. These are thought to contribute to improvements in various psychiatric conditions including mood disorders and substance dependence. This open label exploratory study explored safety, tolerability, physical, mental health and psychedelic effects of three Acacia based formulations in 9 healthy volunteers with prior use of Ayahuasca.

**Method:**

Formulations derived from two Acacia species (1mg/kg DMT and 4mg/kg of harmalas) were tested in a cross-over design in 5 adults; a third formulation (ACL-010) was tested in 4 adults at two dosages (1mg/kg DMT and 4mg/kg of harmalas, and then 1.4mg/kg DMT and 5.6mg of harmalas).

**Results:**

All formulations had a good safety profile. No serious adverse events were reported. Physical examination, vital signs, and pathology revealed no clinically significant changes across the course of the study. The subjective experience of all formulations was generally rated similar to Ayahuasca. Four-week follow-up measures of psychological wellbeing and perceptual effects showed little difference between formulations. The strength and quality of the psychedelic experience elicited with ACL-010 was rated as similar or more beneficial than Ayahuasca.

**Discussion:**

Our results indicate DMT formulations derived from the Acacia species represent a feasible alternative to traditional Ayahuasca for future clinical trials and possibly clinical contexts. The small sample size and open label design limit generalizability of results.

**Clinical trial registration:**

https://www.anzctr.org.au/Trial/Registration/TrialReview.aspx?id=384191&isReview=true, identifier ACTRN12622001315707.

## Introduction

Traditionally, plant-based formulations consisting of the active constituents *N,N* -dimethyltryptamine (DMT) and harmala alkaloids have been used for centuries, known more broadly as ‘Ayahuasca’ (1). This traditional plant-based preparation has been used in the Amazon basin for hundreds of years for a range of therapeutic and psycho-spiritual effects (2, 3). Ayahuasca was also adopted as a religious sacrament by several Brazilian syncretic religions in the 1930’s, and these have now expanded internationally to Europe, North America, and Australia (3–6).

Ayahuasca has dramatically increased in popularity since the turn of the millennium, with increasing numbers of tourists visiting the Amazonas in search of therapeutic or spiritual effects (7). Use of the brew outside the Amazon in neo-shamanic ceremonies has also increased in popularity, with various sources of harmala alkaloids and DMT being used across the globe (4, 5, 8). Vast amounts of anecdotal evidence now exist describing the healing effects of the brew, leading researchers to examine the therapeutic potential of DMT-harmala concoctions (9). In Australia, this use extends to native Acacia-based Ayahuasca formulations (to provide the DMT content), which may be used in combination with *Banisteriopsis caapi* (the Ayahuasca vine) or *Peganum harmala* (Harmel) to provide the harmala compounds.

The psychoactive compounds of Ayahuasca are regarded as DMT, which is present in traditional brews containing *Psychotria viridis* or other related species, and three main β-carbolines (harmine, harmaline and tetrahydroharmine) which are found in *Banisteriopsis caapi*. These β-carbolines are reversible inhibitors of monoamine oxidase (MAOI), while the tetrahydroharmine is also an inhibitor of serotonin reuptake (10). The MAOI function of β-carbolines inhibits DMT degradation in the gastrointestinal system allowing this substance to reach the brain, where it activates serotonergic pathways via 5HT 2A receptor interaction (11). Additionally, research has indicated that harmine could have a central role in Ayahuasca’s anti-addictive effects, including reducing recidivism to alcohol, cocaine and methamphetamine potentially due to MAO-A inhibition, Sigma-1 activity, and neurogenesis promotion (12–14).

A range of potential psychotherapeutic mechanisms have been proposed for ayahuasca, listed below. Combined, these are thought to contribute to improvements in various psychiatric conditions via: Decentering: the ability to observe one’s own thoughts and feelings in a detached, more objective manner (15, 16); Certain mindfulness capabilities: acceptance (non-judgmental and non-reactive processing) and improved observation (17–19); Cognitive flexibility: mental ability to adjust to activity and content (20, 21); Emotional regulation (15); Experiential acceptance (22, 23). While ayahuasca and other classic psychedelics such as psilocybin and LSD share similar effects (e.g. altered perceptual and visual effects and ego dissolution), ayahuasca is more commonly associated with intense emotional catharsis, somatic purging (e.g. vomiting), and vivid visionary experiences potentially involving spiritual themes.

Use of DMT-Harmala formulas have been linked to changes in a range of personality traits. Increases in agreeableness and openness as well as decreases in neuroticism have been observed, with reductions in neuroticism correlating with the subjective intensity of the mystical experience (24, 25). Ayahuasca-induced reductions in grief have been linked to increases in acceptance and the ability to psychologically decenter (26).

Psychedelic agents are however recognized to potentially elicit a range of adverse events (AEs) (27, 28) which are usually transient, including headaches, nausea and possible emesis, anxiety, panic, or agitation, alterations in blood pressure or heart rate, and in rare cases increases in suicidality. Psycho-perceptual changes such as visual/auditory/kinesthetic hallucinations, time distortions, and feelings of awe and transcendent spiritual experiences are considered to not be AEs as such, with data from use in naturalistic settings showing that such mystical experiences are directly related to therapeutic outcomes (29–31).

Recently there has been interest in the development of DMT-harmala preparations as standardized pharmaceutical grade medicines for the clinical treatment of mental health disorders (32). For botanically derived medicines, the use of alternative plant sources of DMT and harmala alkaloids may provide a more scalable option - potentially growing faster and yielding higher concentrations of the active alkaloids. This approach also helps prevent the depletion of Indigenous plant stocks in South America, which is a conservation concern.

The primary purpose of our study was to test the safety, tolerability, and psychedelic effects of three Acacia-based, purified and standardized DMT and harmala alkaloid preparations in healthy volunteers who had experience with Ayahuasca, while also evaluating secondary psychological outcomes. We studied two differing Australian native *Acacia* spp. (both classified within Acacia section Juliflorae, a taxonomic grouping within the genus Acacia sensu stricto), providing the DMT component, in combination with *Peganum harmala* which is a prolifically growing shrub in the Middle East and Asia, providing the β-carbolines. The aim was to explore if any differing safety or psychoactive effects occurred between the species (which have slightly different alkaloidal profiles; assayed via HPLC), and if any additional changes occurred from further purifying the active constituents. Our findings will inform a planned Phase 1 pharmacokinetics/pharmacodynamic study, and a randomized controlled trial involving participants with major depressive disorder and alcohol use disorder.

## Materials and methods

### Trial oversight

The study protocol, Patient Information and Consent Form (PICF), Investigator Brochure, and subsequent amendments were approved by the relevant institutional Human Research Ethics Committee (HREC 118/22). The conduct of this study was in compliance with the approved protocol, and Good Clinical Practice guidelines. The trial was registered with the Australian New Zealand Clinical Trials Register (ACTRN 12622001315707).

In Australia, DMT and harmala alkaloids are prohibited substances [Schedule 9 (S9)] that, by law, may only be used for research purposes. Permits for individual trial participants were granted by Medicines and Poisons Regulation, Department of Health, Victoria, Australia.

This report conforms to the CONSORT reporting guidelines for non-randomized pilot and feasibility studies (33–35).

### General study design

**Figure 1:**
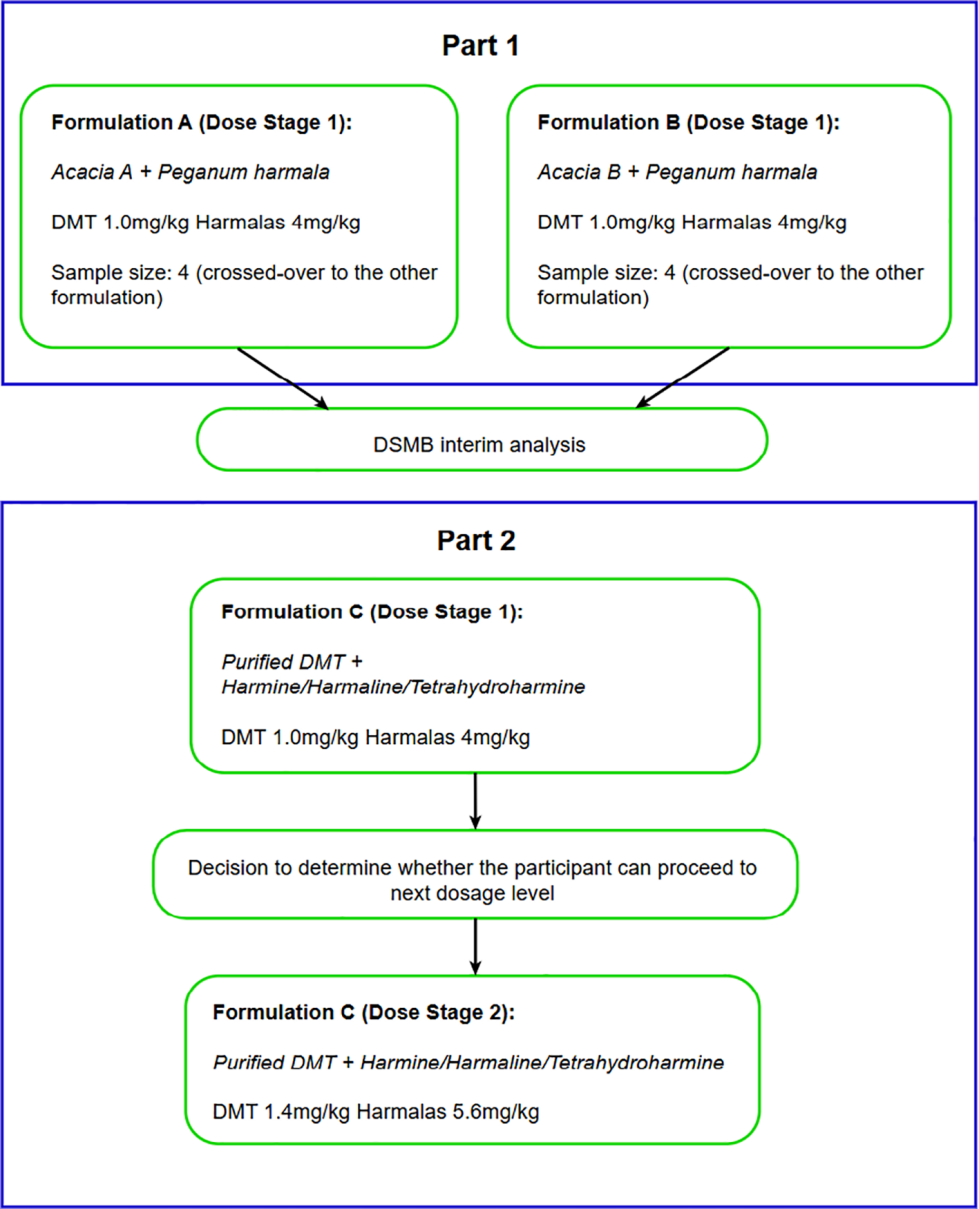
Overall study and design plan.

This was an exploratory pilot study to test three purified and standardized Australian native Acacia-based formulations of DMT and harmala alkaloids in 9 healthy participants with prior use of oral liquid DMT-harmala preparations, such as ayahuasca. The aim of the study was to provide pilot data on formulation, dose, safety, tolerability and subjective effects of study medication to inform pharmacokinetic studies and a planned Phase 2 study. **Figure 1** shows the overall study design and plan for the current study. In Part 1 of the study two formulations of the DMT were studied, derived from different Acacia species, (standardized to 1mg/kg) and harmalas: harmine, harmaline, tetrahydroharmine (standardized collectively to 4mg/kg) formulation (Acacia A + Peganum versus Acacia B + Peganum). Participants were crossed over to experience both formulations (A and B) in an open label manner (9 treatment sessions in total - including one participant withdrawal after the first session). Participants, therapists and researchers were blinded as to the order in which the formulations were administered. In Part 2 of the study, after an interim data analysis, a third formulation was developed, which was derived from Acacia B source in combination with the Peganum component which achieved a purity of >90% DMT and >90% harmala alkaloids (Formulation C; ACL-010). Four participants were given Formulation C at 1mg/kg DMT and 4mg/kg harmalas in the first dosing session, being titrated to 1.4mg/kg DMT and 5.6mg/kg harmalas in the subsequent session (See **Figure 1**). Treatment sessions were a minimum of seven (7) days apart. In both treatment sessions participants were attended by a therapeutic dyad consisting of a psychiatrist (male) and clinical psychologist (female) both with extensive experience in psychedelic assisted psychotherapy.

### Study setting

Dosing treatment sessions were conducted in specially prepared treatment rooms. Attention was paid to the comfort and aesthetic qualities of the room including the use of subdued lighting, a carefully selected music list, plants and aromatherapy. After dosing, participants were encouraged to lie or sit on the bed, wear eyeshades, and listen to the music list via noise cancelling headphones. They were free to move about the room and could remove headphones and eyeshades if not required.

### Primary and secondary outcomes

#### Primary outcomes

Safety and tolerability was assessed based on Adverse Events (AEs)/Serious Adverse Events (SAEs) post study recruitment; vital sign data including body temperature, heart rate, respiratory rate, and blood pressure during and after the treatment session. Integration Difficulties Scale (36) was used to assess any negative mental health effects of the treatment (1 week post-treatment). Psychedelic effects were assessed using the Mystical Experiences Questionnaire (MEQ) (37).

#### Secondary outcomes

Acute subjective effects of the psychedelic experience were assessed via the Five Dimensions of Altered States of Consciousness (5D-ASC) scale (38). The modified Short Index of Mystical Orientation (SIMO) (36, 39) measured the intensity of the participant’s acute mystical experience, and an additional single item was added measuring acute extreme fear “Feeling of immense fear…” on a 10-point scale (36). Visual analogue scales (40) acutely assessed mood and anxiety.

To assess four week follow-up mental health effects the following scales were employed: DASS-21 (41), PANAS – SF (42); Kessler-10 (K-10) scale (43). Persisting Effects Questionnaire (PEQ) (44) was employed to assess chronic impact of the treatment. Insomnia Severity Index (ISI) (45) assessed the nature, severity, and impact of insomnia. Temporal Experience of Pleasure Scale (TEPS) (46) measured individual trait dispositions in both anticipatory and consummatory experiences of pleasure. Personal Insights Questionnaire (PIQ) (47) reported the number personal insights experienced. The Integration Difficulties Scale (IDS) (36) assessed integration-related feelings and experiences. The Ayahuasca Preparation and Support Scale (36) rated preparation and support prior and during treatment sessions.

### Eligibility criteria

#### Inclusions

Male or female; aged 25 to 75 years; mental health professionals with an expressed interest in psychedelic assisted psychotherapy; medically and psychiatrically healthy as adjudicated by the investigator based on physical exam and MINI (DSM-5) (48) psychiatric interview; previously tried DMT-harmalas (i.e. Ayahuasca; but not in the last one month); weight between 50kg and 95kg; BMI of 18 to 32; availability of a friend or family member to assist with transport after the active drug session; willing to adhere to dietary requirements prior to the active treatment session including abstinence from alcohol; willing to take adequate contraception measures during the study.

#### Exclusion

History of psychosis: past or present diagnosis of bipolar disorder, schizophrenia, or schizoaffective disorder; Family history of psychosis: past or present diagnosis of bipolar disorder type 1 in first degree relative, or schizophrenia, or schizoaffective disorder in first or second degree relative; current suicidality or history of suicide attempt; current psychiatric disorder diagnosis; daily/weekly high-risk alcohol use [AUDIT: Alcohol Use Disorders Identification Test (49)]; Use of any psychoactive medication (e.g., a selective serotonin reuptake inhibitor such as paroxetine or citalopram), haloperidol, any medication with Monoamine oxidase activity (such as isocarboxazid, phenelzine, selegiline or tranylcypromine, linezolid, and methylene blue), or any drug that has been indicated as a potential precipitative agent for serotonin syndrome within 28 days prior to study drug administration and through to the end of study; currently taking any other regular medication, including: opioids, antihistamines, anticonvulsants, amphetamines, Kava, and St John’s wort; used an hallucinogen in the month prior to treatment session (a one-month wash-out is acceptable); Use of any recreational drug within the past month (e.g. amphetamines, opioids); smoking/using nicotine; substance/alcohol use disorder; history of Hallucinogen Persisting Perception Disorder (HPPD); serious medical condition e.g., cardiovascular, metabolic, neurological, respiratory, oncological, hematological disorder; serious ECG abnormality; serious abnormal hematology or electrolyte, renal or liver test result (indicated by screening aspartate aminotransferase (AST) or alanine aminotransferase (ALT) ≥2 or total bilirubin ≥1.5 x upper limit of normal (ULN), which remains above these limits if retested) in the previous 12 months (as provided by their GP or SVHM); females who were pregnant, nursing, or trying to become pregnant (pregnancy test provided); not agreeing to fasting from midnight prior to the Dose Day sessions until the afternoon of that treatment day; participation in another clinical study involving investigational study treatment within 30 days or 5 half-lives, whichever was longer, prior to screening.

### Investigational product

All formulations were orally delivered consisting of DMT to harmala alkaloids at a 1:4 ratio, respectively. The 3 principle harmala alkaloids were harmine, harmaline, and tetrahydroharmine, which were provided at a set ratio with relatively low levels of harmaline (the specific DMT to individual harmalas ratio is proprietary information). The dosage is based on the doses used in previous oral Ayahuasca/DMT studies (50–52).

The formulations used in Part 1 of this research (Formulas A and B) were produced at NICM Health Research Institute, Western Sydney University via the following general process:1) Initially a horticulturist confirmed the plant materials (*Peganum harmala* seeds, and phyllodes and thin stems from two Australian native Acacia spp. [Formula A an Acacia species sourced from a private orchard from Northern New South Wales in Australia; Formula B *A.acuminata* sourced from a commercial source in Western Australia]. Note that the Formula A species is not disclosed due to concerns over potential wild harvesting; 2) Plant material was dried and milled; 3) Extraction of the plant constituents occurred via a heptane/ethanol extraction, before the application of a proprietary method to create a two final water solution extraction. A pH decrease was also applied to facilitate a higher yield of the active constituents (i.e. DMT and harmala alkaloids), while reducing the level of un-needed constituents; 4) The solute was then evaporated; 5) The dried powder was then encapsulated in a compounding pharmacy based in Melbourne; 6) A sample of capsules was then tested at partner labs to ensure standardization of the constituents and also to confirm the presence of no contaminants or obvious extraneous toxins (e.g. aflatoxins). The alkaloidal levels being ~5% and ~13% DMT for *Acacia* spp. A and *Acacia* spp. B (*A.acuminata*), respectively, and ~48% for the harmalas for the first formulas, with the remaining constituents being other plant constituents which were less than 2% individually (as revealed via HPLC assay).

Formulation C used in Part 2 of the study was developed in concert with CSIRO Australia and manufactured at NICM Health Research Institute, Western Sydney University. Formulation C provided a standardized combination of DMT (>90% purity) and harmala alkaloids (>90% purity) from *Acacia acuminata.* and *Peganum harmala*. The plant material was processed and manufactured via a similar process to the first formulations with additional purification steps. 

In Part 1 of the study, formulations A and B were administered at a dosage of 1.0.mg/kg of DMT and 4mg/kg of total harmalas. In Part 2 of the study, ALC-010 was administered at 1.0.mg/kg of DMT and 4mg/kg of total harmala in the first dosing session and titrated to 1.4mg//kg of DMT and 5.6mg of total harmala alkaloids in the second dosing session if deemed suitable.

Formulation A was derived from an Eastern Australian *Acacia* spp. and *Peganum harmala*. Each capsule contained 18 mg and 5 mg of DMT, along with 108 mg and 22 mg of harmala alkaloids.

Formulation B was derived from *Acacia acuminata* and *Peganum harmala*, delivering 22.5 mg and 6.5 mg of DMT per capsule, in addition to 108 mg and 22 mg of harmala alkaloids.

Formulation C (designated as ALC-010) was a purified DMT-harmala extraction derived from *Acacia acuminata* and *Peganum harmala*, with each capsule containing 20 mg and 5 mg of DMT, as well as 80 mg and 20 mg of harmala alkaloids.

Standardized DMT doses used in this study were at the upper end of those reported in other clinical studies using oral DMT-harmala preparations. Lower standardized mg/kg doses of DMT (0.3-0.4mg/kg) were reported by Palhano-Fontes et al. (53) and Lanaro et al. (54). Several studies have reported using DMT 1mg/kg (55, 56), while standardized DMT doses of 1.76mg/kg were reported by and Zeifmann et al. (57), and total DMT doses of 96mg-160mg by Sanches et al (58).

Stability data provided by NICM Health Research Institute at Western Sydney University showed that all formulations were stable (within 10% specification deviation) for DMT and harmala constituent levels via post-study HPLC analysis.

### Recruitment

**Figure 2:**
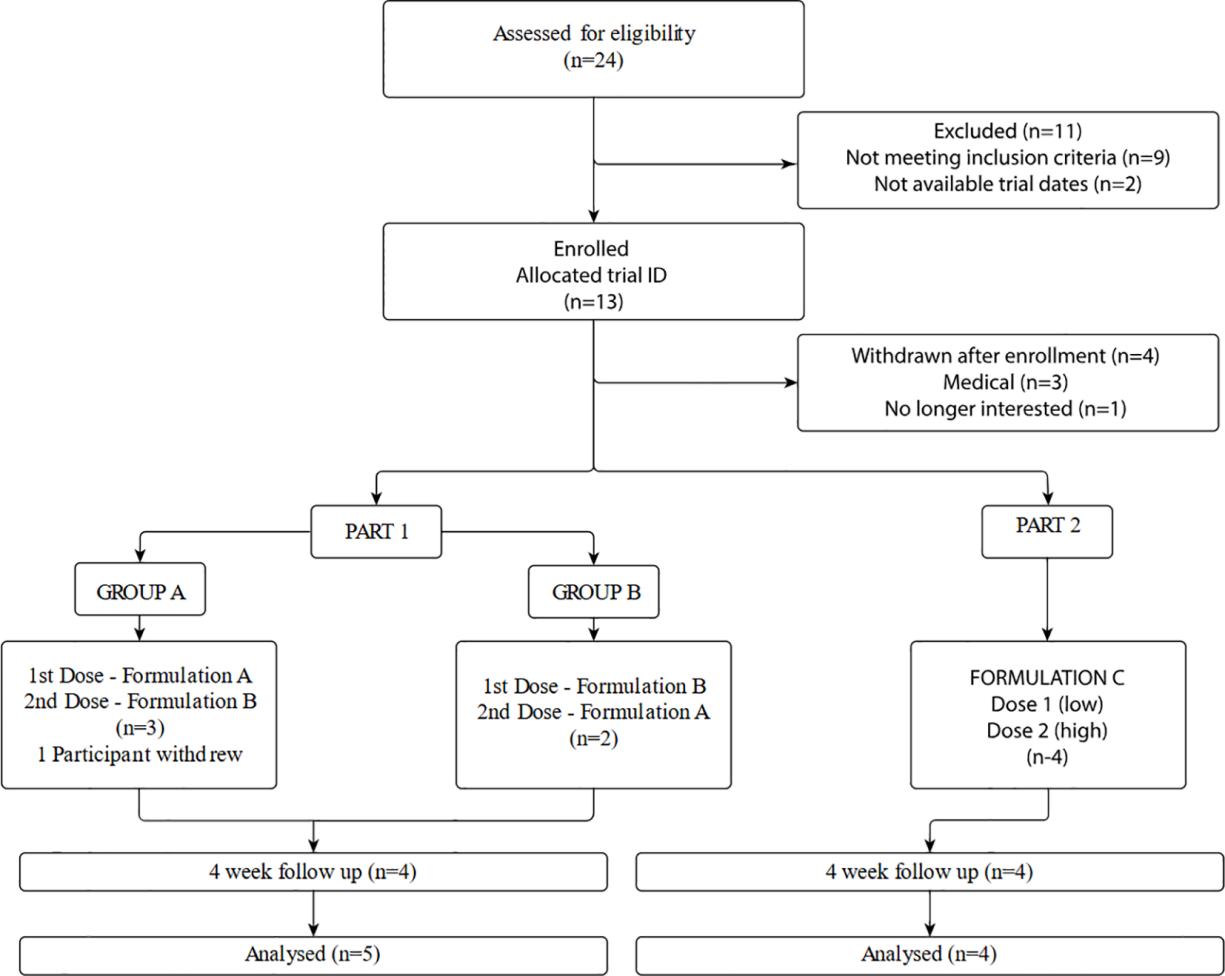
Trial enrolment and disposition.

Recruitment took place between December 2022 and November 2023. Potential participants were recruited from professional networks, via word of mouth, and self-referral. Twenty-four participants were screened for eligibility by phone. Participants who were deemed to meet broad eligibility criteria attended an in-person screening visit for clinical assessment and informed consent. Reasons for exclusion and subsequent trial enrolment and disposition are shown in **Figure 2**.

### Trial procedures

#### Screening and baseline assessment

During the in-person screening process a detailed physical and mental health history, and substance use history was taken including MINI-Plus psychiatric interview, the Alcohol Use Disorders Identification (AUDIT) and a general physical examination. This involved assessment of the general medical exclusion criteria, including blood pressure, heart rate, height, weight, body temperature, an ECG and blood screening (including pregnancy test if female). After review of the Participant Informed Consent Form (PICF) the participant was asked to sign the consent form and scheduled for their preparatory psychotherapy session. Participants were required to self-complete baseline psychometric assessments: Depression, Anxiety and Stress Rating Scale – 21 questions [DASS-21(41)]; Insomnia Severity Index [ISI (45)] – modified; Temporal Experience of Pleasure Scale [TEPS (46)]; Kessler - K10 (43); Positive and Negative Affect Schedule – Short Form [PANAS- SF (42)]. Participants were allocated to receive Formula A or Formula B in the first treatment session, in a cross-over design.

#### Preparatory session

The preparatory session lasted approximately 90–120 minutes with both treating therapists. During these sessions, the therapists provided psychoeducation to prepare the participant for the dosing session. The participant and therapists discussed what would happen during the session including relaying some of the commonly experienced effects of DMT-harmala medicines and the participants’ expectations for the dosing session. Therapists also inquired about any possible changes in the participant’s health to confirm that the participant met eligibility criteria. Participants were oriented to the optional use of therapeutic touch and given the opportunity to provide informed consent to include touch during their session, having the choice to rescind consent at any time. Participants were also given instructions regarding dietary restrictions and fasting requirements prior to treatment, arrival time, suitable clothing, use of an eye mask and headphones, and the music playlist. Participants were advised that in case of extreme persistent agitation an oral, intravenous, or intramuscular delivered sedative and antipsychotic combination (lorazepam and haloperidol) would be administered (from the psychiatrist or physician attending the session).

#### Dosing treatment (2 sessions)

On the day of the dosing treatment session the participant arrived approximately 1 hour prior to dose administration (aiming to arrive at 9am). Therapists reviewed procedures for the experimental session with the participant and discussed any participant concerns. Participants were asked to reconfirm consent in writing and confirm that they had fasted from the midnight and had not taken any excluded herbal supplements, contraindicated foods or medications (prescribed or over-the-counter). Participants were required to self-complete a visual analogue scale (VAS) of mood and anxiety and the PANAS. Participants’ blood pressure (BP) and heart rate (HR) were recorded. Approximately 1 hour after arrival participants were provided the oral dose capsules with a glass of water and instructed to take one harmala capsule followed by one DMT capsule in an alternating manner (to ensure a blended consumption). A 15-minute guided body scan and breathing audio track was played after which the music soundtrack commenced.

At least one of the trial therapists remained with the participant throughout the entire session (both therapists remain with the participant for most of the time, with the opportunity for short breaks) and were available for psychological and medical support during that time. This included a non-directive participant led approach to support the participant in exploring whatever psychological experience was emerging.

Cardiovascular monitoring (heart rate and blood pressure) was conducted at 90 minutes and again between 4 hours and 8 hours (based on when the participant’s psychedelic experience had waned; or assessed more regularly if needed), as well as ongoing monitoring of the participant psychiatric state throughout the dose sessions.

The session ended if all medical and psychiatric parameters were acceptable and the participant was alert, ambulatory, and psychologically stable. Prior to leaving at the end of the session, participants completed various measures assessing different aspects of their experience during the session, including: the Five Dimensions of Altered States of Consciousness scale [5D-ASC (38)], Mystical Experience Questionnaire [MEQ (37)] and VAS assessment of mood and anxiety.

The participant support person (close friend or family member) provided transportation to their residence. Therapists remained available to speak with participants for 24 hours after the treatment session via a study mobile phone number.

#### Post-treatment next day follow-up (2 sessions)

Participants were contacted by phone by the research coordinator the day following each treatment session. They were assessed for any AEs, any use of concomitant medications, and provided a general debrief. The PANAS was emailed to them to complete that day online. Participants were encouraged to use their dream diary. Participants were requested to inform study staff of any emergent AEs or inter-current illnesses. They were also reminded of all study restrictions.

#### One-week post-treatment follow-up assessments (2 assessments)

One week post-treatment participants were emailed a link for online self completion of the following assessments: VAS; Persisting Effects Questionnaire (PEQ); PANAS; TEPS; K-10; DASS-21; ISI- modified; Personal Insights Questionnaire (PIQ); Integration Difficulties Scale (IDS); Preparation & Support assessment (PIS); The modified Short-Index of Mystical Orientation (SIMO).

#### Integration (2 sessions)

The integration psychotherapy session was scheduled approximately one week after each dosing session and was conducted either in-person, or via videoconferencing. The participant was encouraged to discuss their insights, feelings and experiences during and after the session. The therapists supported the participant to process any residual psychological distress they were experiencing. The therapists were supportive, validating the participant’s experience, to facilitate emotional processing, allow exploration of content, and consolidate any therapeutic insights gained. Therapists assessed the participant’s mental health and the presence of any remaining reactions during integrative psychotherapy immediately after the dosing session. The participant was reminded that the therapists would be available for support outside the scheduled integration session via phone, telehealth, or in person by arrangement if extra support was required.

#### Four-week post-treatment follow-up and qualitative interview

Participants were contacted by phone by the research coordinator four weeks post their second treatment session. They were assessed for any emergent AEs, any use of concomitant medications, and a general debrief. Participants were sent an email link to complete Persisting Effects Questionnaire (PEQ); VAS; PANAS; TEPS; K-10; DASS-21; ISI- modified; Personal Insights Questionnaire (PIQ); Integration Difficulties Scale (IDS); Preparation & Support assessment (PIS); open-ended free text items.

A 60-minute qualitative interview was conducted approximately 4 weeks after the final treatment session by a post-graduate student with protocol specific and GCP training. Therapists were also interviewed to assess their experiences of conducting the treatment model. Results of the qualitative research will be published separately.

A detailed schedule of procedures and assessments is included as **Appendix 1**.

### Data preparation and analysis

Baseline demographic and background variables are summarized for all participants. For categorical variables, frequencies and percentages are provided. For continuous variables, descriptive statistics including the sample size, mean, median, standard deviation and range, are presented. Continuous variables are summarized descriptively providing, where applicable, the number of participants, mean, standard deviation (SD), median, interquartile range (IQR), minimum (min) and maximum (max). Individual (absolute and change from baseline) and summary blood pressures, heart rate, respiratory rate, and body temperature and oxygen saturation are presented using descriptive statistics including mean, median, and standard deviation and range (min and max) as appropriate. AEs are summarized from each participant’s Adverse Event log by total individual number for each individual type of AE. The AE s on the Adverse Event log and the rating of severity is described. Serious Adverse Events (SAEs) and SAEs, drug medication-related AEs and serious drug-related AEs are also summarized.

## Results

Given the small number of participants in this study and the inter-individual variation in responses, application of statistical analyses to the data is not appropriate. Aggregated scores and individual responses are presented where appropriate in tabular and graphic form.

### Participant characteristics

Participant characteristics at baseline are shown in **Table 1** for all participants who received at least one dose of the trial medication. Participants were five male and four female, aged 32 to 54 years (mean 40.6, SD 7.5 years). All were tertiary educated. Participants had previously used Ayahuasca on average 2.2 times (SD 1.5; range 1–5 times). The mean duration between most recent use and first trial dose was 5.3 years (SD8.2 years; range of 0.2 to 26.4 years).

**Table 1:** Participant characteristics at baseline (n=9).

Characteristic	Category / descriptive statistic	Participants
Gender	Female / male *(n, %)*	4 (44.4) / 5 (55.6)
Age (years)	*Mean (standard deviation)*	40.6 (7.5)
	*Median (interquartile range)*	39.2 (36.0 – 45.8)
	*Range (minimum – maximum)*	31.8 – 53.5
Country of birth	Australia *(n, %)*	7 (77.8)
	Other (South Africa; Singapore) *(n, %)*	2 (22.2)
Aboriginality	Non Aboriginal / Torres Strait Islander *(n, %)*	9 (100)
Highest level of education	Post-graduate *(n, %)*	9 (100)
Ayahuasca Use History – *number of sessions*	*Mean (standard deviation)*	2.2 (1.5)
	*Median (interquartile range)*	2 (1 – 3)
	*Range (minimum – maximum)*	1 – 5
Ayahuasca Use History – *date of first use*	AEOS_1	2016
	AEOS_2	2016
	AEOS_3	2013
	AEOS_6	Jan-23
	AEOS_7	Aug-18
	AEOS_9	Jul-23
	AEOS_10	1997
	AEOS_11	Nov-19
	AEOS_13	Aug-19
Ayahuasca Use History – *date of most recent use*	AEOS_1	2018
	AEOS_2	Late 2020
	AEOS_3	Nov-22
	AEOS_6	Jan-23
	AEOS_7	Aug-18
	AEOS_9	Jul-23
	AEOS_10	1997
	AEOS_11	Nov-19
	AEOS_13	Aug-19
Ayahuasca Use History – *duration between most recent use (before trial) and first dose of trial (years)*	AEOS_1	4.7
	AEOS_2	2.4
	AEOS_3	0.5
	AEOS_6	0.2
	AEOS_7	4.8
	AEOS_9	0.3
	AEOS_10	26.4
	AEOS_11	4
	AEOS_13	4.3
	*Mean (standard deviation)*	5.3 (8.2)
	*Median (interquartile range)*	4.0 (0.5 – 4.7)
	*Range (minimum – maximum)*	0.2 – 26.4

### Primary outcomes

#### All adverse events/ serious adverse events

All nine participants (100%) who received at least one dose of trial medication reported at least one Adverse Event (AE) from the signing of consent through to the end of the trial.

There were no serious adverse events (SAEs) that occurred during this study.

Of the non-serious adverse events, a total of 71 AEs occurred across the nine participants throughout the study. Three of these events were classified as severe (Flu Type A; Chest infection secondary to Flu; Agitation). Another 16 events were classified as moderate severity (urinary tract infection; insomnia; irritability x 2; panic attack; perceptual disturbance; nightmare; anxiety x 2; depressed mood; delusion; suicidal ideation; aggressive behavior; myalgia; pain; scar). The remaining 52 AEs were classified as mild severity.

#### Study medication related adverse events

Eight participants reported at least one AE that was considered to be related to the study medication. Of all AEs, 55 in total were considered to be study medication-related (78%, 55/71) (see **Table 2**). The most frequently occurring study medication-related AEs were adverse physical effects (69%, 38/55), followed by adverse mental health effects (31%, 17/55). The two most common physical AEs were “nausea” (n=11) and “headache” (n=7), while vomiting/retching occurred in 2 out of 9 participants. The most common mental health effect was anxiety (4/17). Of the 17 mental health effects, 11 events occurred when one participant received the higher dose of ACL-010 Formulation C (n=11). The high dose was well-tolerated by the other 3 participants. See **Table 2**.

**Table 2:** Number of study medication-related adverse events (n=55), total numbers and split by most recent study medication formula.

Adverse event (preferred term defined in MedDRA*)	Total number (across both formulations)	Participants 1, 3, 6, 7		Participants 9, 10, 11, 13	
		Most recent formulation administered			
		Formula A	Formula B	Formula C (low dose)	Formula C (high dose)
Number of participants with > 1 event	8 of 9 (88.9%)	4 of 5 (80%)	4 of 4 (100%)	4 of 4 (100%)	4 of 4 (100%)
Adverse physical effects	38	6	10	11	11
General symptom adverse physical effects					
Nausea	11	2	3	3	3
Headache	7	1	3	2	1
Vomiting	3	.	.	1	2
Stomach cramps^1^	2	1	1	.	.
Insomnia	2	.	1	1	.
Abdominal pain	1	.	1	.	.
Fatigue	1	.	1	.	.
Hunger	1	1	.	.	.
Compensatory sweating	1	–	–	1	–
Tachycardia^2^	1	–	–	1	–
Tremor	1	–	–	–	1
Skin abrasions^3^	1	–	–	–	1
Pain^3^	1	–	–	–	1
Scar^3^	1	–	–	–	1
Neurological adverse physical effects					
Brain fog	3	1	–	1	1
Paresthesia	1	–	–	1	–
Adverse mental health effects	17	2	1	3	11
Altered perception adverse mental health effects					
Hallucination, visual^4^	1	1	.	.	.
Perceptual disturbance^5^	2	.	.	1	1
Emotional-cognitive adverse mental health effects					
Anxiety	4	1	.	1	2
Irritability	1	.	1	.	.
Panic attack^6^	1	.	.	.	1
Nightmare^6^	1	.	.	.	1
Depressed mood^6^	1	.	.	.	1
Delusion^6^	1	.	.	.	1
Suicidal ideation^6^	1	.	.	.	1
Agitation^6^	1	.	.	.	1
Aggressive behaviour^6^	1	.	.	.	1
Arthromyalgical adverse physical effects (MR)					
Myalgia	2	.	.	1	1
Total	55	8	11	14	22

MedDRA, Medical Dictionary for Regulatory Activities.

*1) Therapist described these ‘stomach cramps’ as being different from ‘abdominal pain’.

*2) The event of ‘tachycardia’ was a brief increase in heart rate.

*3) These 3 adverse events were due to the one incident of the participant rubbing their head on carpet, causing a slight skin abrasion, followed by 24 hours of moderate severity pain, followed by a scar whilst the abrasion healed.

*4) Therapist described this Hallucination, visual event as a “closed eye visual”. It occurred 4 days post dose (formula A *“Recurrence of visuals from second dose when eyes closed. Not unpleasant, very subtle … this occurred during another altered state exp”.*

*5) Two events were classified as a perceptual disturbance. An event of “disembodied”, and a “feeling disconnected or alone”.

*6 ) All events occurred in one participant following dose 2 of formula C (high dose).

Of the 55 AEs considered to be study medication-related, most had a stop date during the trial (n=53), with 2 events ongoing at the end of the trial (skin abrasion, scar). These two events were related and were effectively a “carpet burn” caused from repeated rubbing on the carpet of the treatment room. Thirty-nine AEs stopped the same day as the event had started, and eight the following day, 37 of 55 AEs did not require any medication / intervention with each event resolving itself. The median duration was 4.8 hours with an interquartile range of 1.1 to 12.3 hours. The shortest event lasted 15 minutes and the longest event was 19 days (mild headache which resolved without medication).

#### Physical examination, vital signs, and pathology

Physical examinations included the following: general appearance, HEENT (head, ears, eyes, nose and throat), skin, cardiovascular system, respiratory system, gastrointestinal system, nervous system, vital signs and other. Any changes in physical condition were noted by the principal investigator and AEs were recorded in the AE log by the study coordinator and trial clinicians. Most participants recorded no changes in general appearance and physical condition across the duration of the study. One participant suffered a facial abrasion during a treatment session which was recorded as an AE.

Vital signs monitoring included body temperature, heart rate, respiratory rate, oxygen saturation and blood pressure. No clinically significant changes were recorded. Vital signs (absolute values, and changes from baseline) are reported in **Appendix 2**.

There were no significant changes in pathology results for participants over the course of the study. Pathology values are reported in **Appendix 3**.

#### Integration difficulties

Integration Difficulties Scale (IDS) showed that participants reported low rates on integration difficulties across all formulations and timepoints with slightly higher average scores for ACL-010 Formulation C (low and high) See **Table 3A**.

**Table 3A:** Integration Difficulties Scale (IDS) 1 and 4 weeks post treatment.

Integration Difficulties Scale (IDS): is a list of 10 difficult experiences. Participants are asked if these difficulties had increased since their treatment session. Answers included “not at all”, “slightly”, “moderately”, “very much” and “unsure”.							
Outcome measure	Descriptive statistic	Product A (n=5)	Product B (n=4)	A/B (n=4)	Product C (low dose) (n=4)	Product C (high dose) (n=4, unless specified)	
		1 week post T*	1 week post T*	4 week post T*	1 week post T1	1 week post T2	4 week post T2 (n=3)
Count of	Mean (SD)	9.4 (0.9)	9.5 (1.0)	9.5 (0.6)	8.5 (1.9)	8.3 (0.5)	8.7 (0.6)
“not at all”	Min – Max	8 – 10	8 – 10	9 – 10	6 – 10	8 – 9	8 – 9
Count of “slightly”, “moderately” and “very much”	Mean (SD)	0.6 (0.9)	0.5 (1.0)	0.5 (0.6)	1.5 (1.9)	1.8 (0.5)	1.3 (0.6)
	Min – Max	0 – 2	0 – 2	0 – 1	0 – 4	1 – 2	1 – 2
Count of	Mean (SD)	0	0	0	0	0	0
“very much”	Min – Max	–	–	–	–	–	–

#### Mystical experience

On the primary psychometric scale outcome measure, the MEQ (given immediately after each psychedelic experience), the mean total MEQ scores were higher (and within the range of Complete Mystical Experience) for ACL-010 Formulation C (low and high) compared to formulas A and B, and on all MEQ subscales except “Positive Mood”. However, it should be noted that the high SD on all scales indicates high inter-individual variability See **Table 3B**.

**Table 3B:** Mystical Experience Questionnaire (MEQ).

Mystical Experience Questionnaire (MEQ): 6-point rating scale (0 = “none; not at all”, 1 = “so slight cannot decide”, 2 = “slight”, 3 = “Moderate”, 4 = “strong”, and 5 = “extreme (more than ever before in my life)”. The sum of the answers in each subscale are expressed as a percentage of the maximum possible.					
Outcome measure	Descriptive statistic	Product A (n=5)	Product B (n=4)	Product C (low dose) (n=4)	Product C (high dose) (n=4, unless specified)
		Post T*	Post T*	Post T1	Post T2
1. Mystical	Mean (SD)	38.4 (15.7)	47.7 (35.5)	62.3 (36.2)	71 (33.2)
	Minimum - Maximum	18.7 – 56.0	6.7 – 93.3	10.7 – 94.7	24.0 – 100
2. Positive Mood	Mean (SD)	46.7 (24.4)	61.7 (19.3)	67.5 (40.8)	70 (34.7)
	Minimum - Maximum	23.3 – 80.0	40.0 – 86.7	6.7 – 93.3	20.0 – 100
3. Transcendence of Time/Space	Mean (SD)	44.7 (22.9)	55 (28.4)	79.2 (23.8)	85.0 (13.7)
	Minimum - Maximum	16.7 – 70.0	13.3 – 76.7	46.7 – 100	73.3 – 100
4. Ineffability	Mean (SD)	65.3 (17.3)	60 (49.0)	76.7 (34.6)	96.7 (3.9)
	Minimum - Maximum	40.0 – 86.7	0 – 100	26.7 – 100	93.3 – 100
MEQ Total	Mean (SD)	44 (12.4)	53.2 (30.9)	68.2 (33.6)	76.2 (25.8)
	Minimum - Maximum	24.0 – 53.3	14.0 – 89.3	18.7 – 93.3	40.0 – 100

T = treatment session

T1 = treatment session 1

T2 = treatment session 2

T* = treatment session (either 1 or 2 depending on the random order of product A / product B)

Pre T = during the treatment session prior to the administration of the study medication

Post T = during the treatment session at the conclusion of the psychedelic experience (a minimum of 4 hours to 8 hours post dose)

SD = 1 standard deviation

Min = minimum

Max = maximum

### Secondary acute effects

#### Tolerability and differential experience

**Figure 3:**
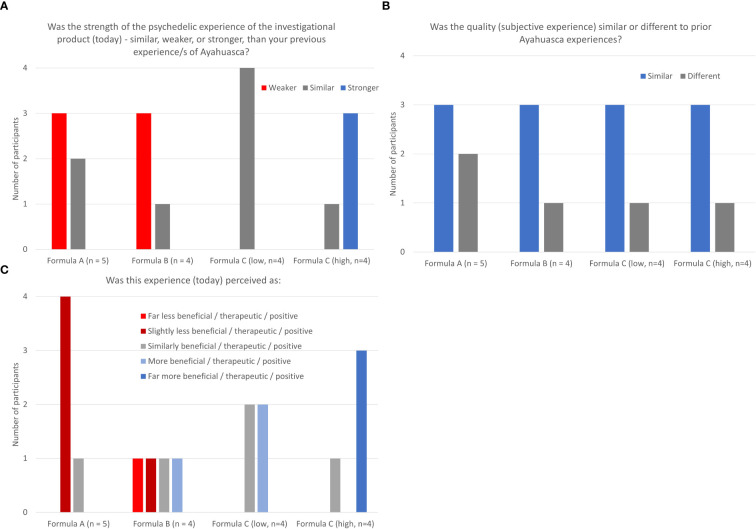
**(A–C)** Tolerability and differential experience: strength, quality, and benefit of psychedelic experience.

At the end of the treatment session trial participants were asked to compare their experience with the trial medication to past experience with traditional Ayahuasca, specifically the strength of the psychedelic experience, the subjective experience (quality), and how beneficial they found the experience. The strength of psychedelic experience of formulations A and B were generally rated as weaker than previous experience with Ayahuasca whereas low dose ACL-010 was rated as similar, with the higher dose version of this formulation being rated as stronger. The subjective experience (quality) of all formulations was generally rated as similar to previous experience with Ayahuasca. Both dose levels of ACL-010 Formulation C were rated as similar or more beneficial than previous experience with Ayahuasca, while Formulations A and B tended to be as less beneficial than previous experience with Ayahuasca See **Figures 3A–C**.

#### Subjective experience

The modified Short Index of Mystical Orientation scores for ACL-010 Formulation C (high) were higher than those for Formulations A, B and C (low). Item 10 “Feeling of immense fear” was given the lowest possible rating by participants after dosing with formulations A and B, however ACL-010 Formula C (high) was rated more highly, and in particular two participants scored 10 (max) and 8 post ACL-010 Formulation C (high). See **Table 3C**. On the Five Dimensions of Altered States of Consciousness (5D-ASC) scale there was a trend on the 11 subscales for higher ratings on ACL-010 (Formulation C), particularly at the higher dose, however there was high inter-individual variability and ratings of the 3 formulas was inconsistent. See **Table 3D**. Both mood and anxiety scores on the VAS were generally relatively low (below 30/100) at each time point but with some inter- and intra-participant variability. Mood scores were generally lower than anxiety scores See **Table 3E**.

**Table 3C:** Acute effect measures: Short-Index of Mystical Orientation (SIMO).

Short-Index of Mystical Orientation (SIMO): 10 items about the participant’s inclination towards mystical experiences and spiritual connectedness. (Responses for each item: 1 = “not at all”; 10 = “very much”).					
Outcome measure	Descriptive statistic	Product A	Product B	Product C (low dose)	Product C (high dose)
		(n=5)	(n=4)	(n=4)	(n=4, unless specified)
		Post T*	Post T*	Post T1	Post T2
Sum of Items 1-9 (maximum = 90)	Mean (SD)	37.2 (13.7)	41.3 (28.1)	53.8 (27.0)	70.8 (20.0)
	Min – Max	23 – 58	10 – 77	31 – 86	41 – 84
Raw score (Item 10: feeling immense fear …)	Mean (SD)	1 (0)	1 (0)	1.8 (1.0)	5.8 (3.9)
	Min - Max	1 – 1	1 – 1	1 – 3	2 – 10

**Table 3D:** 5 Dimensions of Altered States of Consciousness (5D-ASC) scale.

5 Dimensions of Altered States of Consciousness (5D-ASC) scale: Eleven subscales (sum of the answers in each subscale, expressed as a percentage of the maximum possible).					
Outcome measure	Descriptive statistic	Product A	Product B	Product C	Product C
		(n=5)	(n=4)	(low dose)	(high dose)
				(n=4)	(n=4, unless specified)
		Post T*	Post T*	Post T1	Post T2
1. Experience of Unity	Mean (SD)	52.5 (13.2)	51.0 (33.7)	50.4 (31.5)	74.7 (21.8)
	Min - Max	35.4 – 71.8	11.0 – 93.0	17.2 – 89.2	42.8 – 91.6
2. Spiritual Experience	Mean (SD)	55.1 (14.0)	63.6 (36.0)	57.7 (46.5)	70.5 (38.4)
	Min - Max	31.3 – 68.3	18.3 – 96.7	8.3 – 100	14.3 – 100
3. Blissful State	Mean (SD)	57 (15.6)	60.7 (18.4)	41.8 (33.9)	51.2 (26.3)
	Min - Max	33.0 – 75.7	37.3 – 79.0	2.7 – 84.3	22.0 – 85.3
4. Insightfulness	Mean (SD)	54.9 (12.5)	68.3 (21.8)	63.8 (32.6)	69.6 (18.3)
	Min - Max	33.0 – 63.7	36.7 – 86.7	31.7 – 98.3	46.7 – 91.0
5. Disembodiment	Mean (SD)	51.7 (30.6)	54.3 (24.4)	52.8 (19.6)	52.8 (16.2)
	Min - Max	3.0 – 87.7	18.3 – 70.3	31.7 – 72.7	29.0 – 64.0
1. 6. Impaired Control and Cognition	Mean (SD)	24.6 (23.4)	26.2 (22.8)	39.5 (19.6)	50.8 (11.8)
	Min - Max	2.9 – 57.1	0 – 49.7	18.3 – 59.4	40.0 – 67.3
7. Anxiety	Mean (SD)	14.4 (15.2)	21.5 (21.9)	34.7 (29.2)	55.7 (24.8)
	Min - Max	3.0 – 41.0	0 – 49.0	4.0 – 64.2	32.2 – 85.3
8. Complex Imagery	Mean (SD)	50.2 (17.4)	70.6 (25.4)	79.2 (29.1)	88.8 (7.9)
	Min - Max	40.7 – 76.3	37.0 – 95.3	36.0 – 98.0	78.7 – 95.7
9. Elementary Imagery	Mean (SD)	51.7 (29.3)	51.7 (46.9)	79.1 (23.3)	85.6 (14.7)
	Min - Max	27.7 – 92.3	0 - 96.7	49.0 – 98.0	64.3 – 97.0
10. Audio-Visual Synesthesia	Mean (SD)	56.5 (14.8)	65.2 (32.6)	84.1 (19.0)	76.7 (29.3)
	Min - Max	31.0 – 67.7	19.7 – 89.7	57.0 – 100	36.0 – 100
11. Changed Meaning of Percepts	Mean (SD)	36.3 (20.1)	56.7 (40.5)	61.4 (7.3)	66.1 (19.2)
	Min - Max	6.7 – 56.0	0 – 91.3	51.0 – 67.7	40.0 – 84.7

**Table 3E:** Visual Analogue Scale (VAS) for anxiety and mood.

Visual Analogue Scale (VAS) - anxiety: “Note how anxious (on average) you felt over the past 24 hours with the slider on the scale”, (minimum (0) = not at all anxious; maximum (100) = extremely anxious). Visual Analogue Scale (VAS) - mood: “Note how your mood was (on average) over the past 24 hours with the slider on the scale”, (minimum (0) = not at all depressed; maximum (100) = extremely depressed).															
Outcome measure	Descriptive statistic	Product A			Product B			A/B	Product C (low dose)			Product C (high dose)			
		(n=5)			(n=4)			(n=4)	(n=4)			(n=4, unless specified)			
		Pre T*	Post T*	1 week post T*	Pre T*	Post T*	1 week post T*	4 week post T*	Pre T1	Post T1	1 week post T1	Pre T2	Post T2	1 week post T2	4 week post T2
															(n=3)
Anxiety	Mean SD Min - Max	16.2 23.6 1 - 58	12. 14.9 0 - 37	16.8 28.7 1 - 68	24.5 33.6 0 - 74	7.0 4.5 1 - 11	26.3 27.1 3 - 54	14.8 12.4 2 - 30	32.5 20.0 14 - 61	38.8 24.7 11 - 67	20 10.9 12 - 36	24.3 11.6 15 - 40	18.5 11.0 3 - 28	12.5 8.5 6 - 25	23.3 9.3 13 - 31
Mood	Mean SD Min - Max	3 2.3 1 - 6	4.2 5.6 0 - 14	14.4 20.5 0 - 50	5.5 6.0 0 - 14	2.5 4.4 0 - 9	5 4.2 1 - 10	8.5 6.7 3 - 18	22.8 18.5 6 - 49	10 8.6 2 - 22	13 10.8 4 - 27	13.8 14.5 2 - 33	12.3 12.0 2 - 28	27.3 34.8 1 - 78	24.3 24.6 1 - 50

#### Secondary four week follow-up effects

On other outcomes, the Depression Anxiety Stress Scale – 21 (DASS-21), the PANAS, and the Kessler-10 (K-10) ratings across all time points and formulations showed little variation. et al., 20 See **Tables 4A**-**C**. As detailed in **Table 4D**, on the PEQ positive responses for all subscales across all formulations were greater than their negative counterparts. For each of the six positive subscales, responses for Formulations B, C (low and high) tended to be higher than Formulation A. ACL-010 Formulation C (low and high) tended to score more highly on each of the additional parameters “How personally meaningful were the experiences”, “the degree to which the experiences were spiritually significant”, “how psychologically challenging were … the experiences”, “how personally psychologically insightful”, “the degree to which the experiences changed the sense of personal well-being or life satisfaction”. Effects on all subscales tended to continue to the 4-week post last dose assessment.

**Table 4A:** Four-week follow-up effect measures: Depression, Anxiety and Stress Scale (DASS_21).

Depression, Anxiety and Stress Scale (DASS21): Individual responses range from 0 = “did not apply to me at all” to 3 = “most of the time”. For the total of the 7 “depression” items, “normal” depression ranges from 0 – 4. For the total of the 7 “anxiety” items, “normal” anxiety ranges from 0 – 3. For the total of the 7 “stress” items, “normal” stress ranges from 0 to 7.									
Outcome measure	Descriptive statistic		Product A (n=5)	Product B (n=4)	A/B (n=4)		Product C (low dose) (n=4)	Product C (high dose) (n=4, unless specified)	
		Base (n=5)	1 week post T*	1 week post T*	4 week post T*	Base (n=4)	1 week post T1	1 week post T2	4 week post T2 (n=3)
Depression Score	Mean (SD) Min – Max	1.2 (1.3) 0 – 3	0.8 (0.8) 0 – 2	0.5 (0.6) 0 – 1	0.8 (1.0) 0 – 2	0.8 (1.0) 0 – 2	1.0 (1.4) 0 – 3	0.5 (1) 0 - 2	0.7 (1.2) 0 – 2
Anxiety Score	Mean (SD) Min – Max	0 -	0.2 (0.4) 0 – 1	0.8 (1.0) 0 – 2	0.3 (0.5) 0 – 1	0 -	0.3 (0.5) 0 – 1	0.5 (0.6) 0 – 1	0.3 (0.6) 0 – 1
Stress Score	Mean (SD) Min – Max	3.4 (3.0) 0 – 7	3.4 (5.3) 0 – 12	4.3 (3.0) 1 – 8	3.3 (3.8) 0 – 7	2.3 (1.3) 1 – 4	2.8 (1.0) 2 – 4	1.5 (0.6) 1 – 2	1.7 (0.6) 1 – 2

**Table 4B:** Four-week follow-up effect measures: Positive and Negative Affect Scale (PANAS).

Positive Affect and Negative Affect Scale (PANAS): Positive Affect (PA) items are related to pleasurable engagement with the environment, and the Negative Affect (NA) items reflect general distress (e.g. anger, guilt, anxiety). Each item is rated on a 5-point scale ranging from 1=“very slight, or not at all” to 5=“extremely”. The final score is the sum of the ten items for both the positive and negative affect.																	
Outcome measure	Descriptive statistic		Product A (n=5)			Product B (n=4)			A/B (n=4)		Product C (low dose) (n=4)			Product C (high dose) (n=4, unless specified)			
		Base (n=5)	Pre T*	1 day Post T*	1 week post T*	Pre T*	1 day Post T*	1 week post T*	4 week post T*	Base (n=4)	Pre T1	1 day post T1	1 week post T1	Pre T2	1 day post T2	1 week post T2	4 week post T2 (n=3)
Positive Affect (PA)	Mean (SD) Min-Max	38.4 (4.0) 34 - 43	37.4 (2.5) 35 - 40	37.5 (3.7) 32 - 40	33.2 (7.7) 20 - 40	38.5 (5.9) 34 - 47	38.3 (2.9) 35 - 42	37.8 (1.5) 36 - 39	35.8 (9.3) 22 - 42	34 (8.9) 24 - 42	31.5 (4.7) 27 - 36	35.3 (8.6) 23 - 43	35.5 (5.8) 30 - 43	36 (6.3) 29 - 44	n=2* 36.5 (0.7) 36 - 37	36.8 (3.3) 33 - 40	36.7 (3.5) 33 - 40
Negative Affect (NA)	Mean (SD) Min-Max	15.0 (4.0) 11 - 19	15.0 (5.0) 11 - 23	15.6 (5.5) 11 - 24	15.6 (6.3) 10 - 25	17.0 (8.5) 10 - 29	14.8 (4.6) 10 - 20	15.0 (4.2) 11 - 20	15.8 (4.6) 11 - 21	3.3 (1.7) 11 - 15	16.3 (4.9) 12 - 21	16.8 (6.4) 11 - 26	14.0 (3.4) 12 - 19	13.8 (2.2) 11 - 16	n=2* 24.0 (5.7) 20 - 28	13.8 (2.9) 10 - 17	14.0 (1.0) 13 - 15

**Table 4C:** Four-week follow-up effect measures: Kessler-10 (K10).

Kessler-10 (K10): 10 items to identify significant levels of psychological distress. Response to individual items range from 1 = “none of the time” to 5 = “all of the time”.									
Outcome measure	Descriptive statistic		Product A (n=5)	Product B (n=4)	A/B (n=4)		Product C (low dose) (n=4)	Product C (high dose) (n=4, unless specified)	
		Baseline (n=5)	1 week post T*	1 week post T*	4 week post T*	Baseline (n=4)	1 week post T1	1 week post T2	4 week post T2 (n=3)
Total (max=50)	Mean (SD) Min – Max	13.8 (2.7) 10 – 16	14.6 (4.4) 11 – 22	13.8 (2.5) 11 – 17	13.5 (3.4) 10 – 18	13.0 (1.2) 12 – 14	13.3 (1.0) 12 – 14	13.0 (1.6) 11 – 15	12.7 (1.2) 12 – 14

**Table 4D:** Four-week follow-up effect measures: Persisting Effects Questionnaire.

Persisting Effects Questionnaire (PEQ): 140 questions that assess changes in attitudes, moods, behavior, and spiritual experience. Each question is rated on a 6-point scale (0 = “none, not at all” to 5 = “extreme, more than ever before in your life and stronger than 4”). Each of the 6 subscales have a positive and a negative version. Scores are expressed as the percentage of the maximum possible score for each subscale.							
Outcome measure	Descriptive statistic	Product A (n=5)	Product B (n=4)	A/B (n=4)	Product C (low dose) (n=4)	Product C (high dose) (n=4, unless specified)	
		1 week post T*	1 week post T*	4 week post T*	1 week post T1	1 week post T2	4 week post T2 (n=3)
1a) Positive attitudes about life	Mean (SD) Minimum - Maximum	29.2 (10.1) 18.5 – 38.5	48.8 (36.2) 0 – 83.1	38.8 (25.9) 6.2 – 69.2	49.6 (27.0) 18.5 – 78.5	66.2 (24.1) 35.4 – 90.8	62.6 (14.0) 47.7 – 75.4
1b) Negative attitudes about life	Mean (SD) Minimum - Maximum	0.6 (0.8) 0 – 1.5	0.4 (0.8) 0 – 1.5	1.9 (3.8) 0 – 7.7	4.2 (2.3) 1.5 – 6.2	7.3 (4.4) 1.5 – 12.3	4.1 (5.8) 0 – 10.8
2a) Positive attitudes about self	Mean (SD) Minimum - Maximum	24.7 (10.2) 14.5 – 38.2	40.9 (33.9) 0 – 80	35.5 (21.1) 16.4 – 65.5	45.0 (29.6) 5.5 – 74.6	65.5 (16.5) 45.5 – 83.6	59.4 (6.4) 52.7 – 65.5
2b) Negative attitudes about self	Mean (SD) Minimum - Maximum	2.5 (4.7) 0 – 10.9	0.9 (1.1) 0 – 1.8	2.7 (5.5) 0 – 10.9	1.8 (2.6) 0 – 5.5	9.1 (2.6) 7.3 – 12.7	4.2 (1.0) 3.6 – 5.5
3a) Positive mood changes	Mean (SD) Minimum - Maximum	24.9 (7.6) 13.3 – 33.3	38.3 (31.5) 0 – 75.6	32.8 (20.8) 13.3 – 62.2	46.1 (34.4) 0 - 80	53.3 (21.1) 22.2 – 68.9	51.1 (19.4) 28.9 – 64.4
3b) Negative mood changes	Mean (SD) Minimum - Maximum	1.8 (2.4) 0 – 4.4	2.2 (4.4) 0 – 8.9	3.3 (4.3) 0 – 8.9	1.1 (2.2) 0 – 4.4	7.2 (6.4) 0 – 15.6	1.5 (2.6) 0 – 4.4
4a) Altruistic / positive social effect	Mean (SD) Minimum - Maximum	21.8 (12.0) 6.7 – 37.8	42.8 (28.5) 0 – 57.8	36.7 (22.4) 11.1 – 62.2	30.6 (22.0) 0 – 51.1	43.4 (24.4) 17.8 – 71.1	45.2 (9.0) 35.6 – 53.3
4b) Antisocial / negative social effect	Mean (SD) Minimum - Maximum	4.9 (8.5) 0 – 20	3.3 (3.9) 0 – 6.7	5.0 (8.6) 0 – 17.8	1.7 (2.1) 0 – 4.4	7.8 (5.9) 0 – 13.3	2.2 (2.2) 0 – 4.4
5a) Positive behavioral changes	Mean (SD) Minimum - Maximum	24 (8.9) 20 – 40	45 (34.2) 0 – 80	45 (19.1) 20 – 60	40 (46.2) 0 – 80	55 (37.9) 0 – 80	60 (20.0) 40 – 80
5b) Negative behavioral changes	Mean (SD) Minimum - Maximum	0 -	0 -	5 (10) 0 – 20	5 (10) 0 – 20	15.0 (19.1) 0 – 40	6.7 (11.5) 0 – 20
6a) Increased spirituality	Mean (SD) Minimum - Maximum	16.2 (13.9) 0 – 36.4	33.6 (29.7) 0 – 60	29.3 (24.6) 0.9 – 59.1	34.1 (25.0) 0 – 60	41.8 (21.8) 22.7 – 67.3	41.2 (19.8) 19.1 – 57.3
6b) Decreased spirituality	Mean (SD) Minimum - Maximum	1.1 (1.2) 0 – 2.9	0 -	0.7 (1.4) 0 – 2.9	1.0 (1.1) 0 – 1.9	2.4 (3.0) 0 – 6.7	1.0 (1.0) 0 – 1.9
Additional questions about the experiences during the participant’s last session. I) How personally meaningful were the experiences? (1 = no more than routine, everyday experiences to 8 = the single most meaningful experience of my life); II) Indicate the degree to which the experiences were spiritually significant to you? (1 = no more than routine, everyday experiences to 8 = the single most spiritually significant experience of my life; III) How psychologically challenging were the most psychologically challenging portions of the experiences? (1 = no more than routine, everyday experiences to 8 = the single most difficult or challenging experience of my life); IV) How personally psychologically insightful to you were the experiences? (1 = no more than routine, everyday psychologically insightful experiences to 8 = the single most psychologically insightful experience of my life); V) do you believe that the experiences and your contemplation of that experience have led to change in your current sense of personal well-being or life satisfaction? (-3 = decreased very much, 0 = no change, +3 = increased very much).							
I) Personally meaningful	Mean (SD) Minimum - Maximum	3.6 (1.1) 2 – 5	3.8 (2.5) 1 – 7	4.0 (1.4) 3 – 6	5.8 (1.3) 4 – 7	7 (0) 7 – 7	6.7 (0.6) 6 – 7
II) Spiritually significant	Mean (SD) Minimum - Maximum	3.6 (0.9) 3 – 5	3.5 (2.1) 1 – 6	4.3 (1.9) 3 – 7	4.5 (2.6) 1 – 7	7 (0) 7 – 7	7 (0) 7 – 7
III) Psychologically challenging	Mean (SD) Minimum - Maximum	1.8 (0.8) 1 – 3	2.0 (1.2) 1 – 3	3.3 (1.3) 2 – 5	4.8 (1.7) 3 – 7	6.5 (1.3) 5 – 8	6.7 (1.5) 5 - 8
IV) Psychologically insightful	Mean (SD) Minimum - Maximum	2.8 (1.5) 1 - 5	3.8 (2.1) 1 – 6	4.0 (1.6) 2 – 6	5.3 (1.7) 3 – 7	7.3 (0.5) 7 – 8	7.0 (1.0) 6 – 8
V) Change in well-being / life satisfaction	Mean (SD) Minimum - Maximum	0.6 (0.5) 0 – 1	1.3 (1.0) 0 – 2	1.5 (1.3) 0 – 3	1.8 (1.0) 1 – 3	2.0 (0.8) 1 – 3	2.0 (0) 2 – 2

Insomnia Severity Index (ISI) (45) average total scores across all formulations were within the “no clinically significant insomnia” range (noting there was high variability across all formulations on Sleep Quality scores) See **Table 4E**.

**Table 4E:** Four-week follow-up effect measures: Insomnia Severity Index (ISI).

Insomnia Severity Index (ISI) + 2 questions: to assess the nature, severity, and impact of insomnia. Responses to the 7 items are based on a 5-point Likert scale. A total score of 0–7 indicates “no clinically significant insomnia”, 8–14 indicates “subthreshold insomnia”, 15–21 indicates “clinical insomnia (moderate severity)”, and 22–28 indicates “clinical insomnia (severe)”. Sleep Quality: “How would you rate your sleep quality in the last week?” Responses ranged from 0 = Highly satisfactory to 4 = not at all satisfactory. Sleep hours: ”How many hours sleep have you had for the past week?”									
Outcome measure	Descriptive statistic		Product A (n=5)	Product B (n=4)	A/B (n=4)		Product C (low dose) (n=4)	Product C (high dose) (n=4, unless specified)	
		Base (n=5)	1 week post T*	1 week post T*	4 week post T*	Base (n=4)	1 week post T1	1 week post T2	4 week post T2 (n=3)
Total	Mean (SD) Min – Max	5.2 (3.9) 3 – 12	5.8 (5.0) 1 – 14	6.0 (3.9) 1 – 10	5.0 (3.7) 1 – 10	2.8 (1.5) 1 – 4	5.5 (2.1) 3 – 8	4.5 (3.3) 0 – 7	3.7 (1.5) 2 – 5
Sleep Quality	Mean (SD) Min – Max	1.6 (0.9) 1 – 3	1.8 (0.8) 1 – 3	1.5 (0.6) 1 – 2	1.5 (1) 1 – 3	1.0 (0) 1 – 1	1.8 (1.0) 1 – 3	1.3 (0.5) 1 – 2	1.3 (0.6) 1 – 2
Sleep hours (past week)	Mean (SD) Min – Max	47.8 (2.8) 45 – 51	48.0 (5.8) 42 – 56	n=3 49.7 (5.7) 45 – 56	n=3 49.0 (6.1) 45 – 56	49.0 (3.4) 45 – 53	46.8 (3.5) 45 – 52	n=3 49.7 (0.6) 49 – 50	47.3 (2.5) 45 – 50

On other outcome scales, the TEPS data showed that scores across both subscales and total TEPS scores were relatively high for all formulations and time points See **Table 4F**. Personal Insights assessed via the PIQ revealed that the average number of insights experienced was lowest for participants 1 week post dose Formulation A, and greatest for participants 4 weeks post dose ACL-010 Formulation C (high) See **Table 4G**.Finally, on the Ayahuasca Preparation and Support Scale the average of the participant’s responses ranged between 3 (moderately) and 4 (very much). This was consistent across all formulations See **Table 4H**.

**Table4F:** Four-week follow-up effect measures: Temporal Experience of Pleasure Scale (TEPS).

Temporal Experience of Pleasure Scale (TEPS): designed to measure individual trait dispositions in both anticipatory (10 items) and consummatory (8 items) experiences of pleasure. Responses to the 18 items are based on a 6-point Likert scale (1 = “very false” for me to 6 = “very true for me”). Items are averaged in each subscale. Higher scores indicate a stronger tendency to anticipate or experience pleasure.									
Outcome measure	Descriptive statistic		Product A (n=5)	Product B (n=4)	A/B (n=4)		Product C (low dose) (n=4)	Product C (high dose) (n=4, unless specified)	
		Base (n=5)	1 week post T*	1 week post T*	4 week post T*	Base (n=4)	1 week post T1	1 week post T2	4 week post T2 (n=3)
Anticipatory	Mean (SD) Min – Max	4.9 (0.2) 4.5 – 5.0	4.6 (0.9) 3.1 – 5.2	4.6 (0.5) 3.8 – 5.0	4.9 (0.7) 4.0 – 5.6	4.9 (0.3) 4.6 – 5.2	5.1 (0.6) 4.3 – 5.6	5.0 (0.8) 3.8 – 5.7	5.3 (0.6) 4.6 – 5.8
Consummatory	Mean (SD) Min – Max	5.4 (0.2) 5.1 – 5.8	4.9 (1.3) 2.3 – 6.0	5.3 (0.4) 5.0 – 6.0	5.3 (0.5) 4.8 – 5.9	5.1 (0.4) 4.6 – 5.6	5.2 (0.6) 4.6 – 6.0	5.0 (0.7) 4.4 – 6.0	5.0 (0.1) 4.9 – 5.1
Total	Mean (SD) Min – Max	5.1 (0.2) 4.9 – 5.3	4.7 (1.0) 2.9 – 5.4	4.9 (0.4) 4.4 – 5.4	5.1 (0.6) 4.3 – 5.7	5.0 (0.1) 4.9 – 5.1	5.2 (0.4) 4.7 – 5.7	5.0 (0.7) 4.1 – 5.6	5.1 (0.4) 4.7 – 5.5

**Table 4G:** Four-week follow-up effect measures: Personal Insights Questionnaire (PIQ).

Personal Insights Questionnaire (PIQ): the summed number of the 7 personal insights items reported.							
Outcome measure	Descriptive statistic	Product A (n=5)	Product B (n=4)	A/B (n=4)	Product C (low dose) (n=4)	Product C (high dose) (n=4, unless specified)	
		1 week post T*	1 week post T*	4 week post T*	1 week post T1	1 week post T2	4 week post T2 (n=3)
Total count	Mean (SD) Min – Max	3.6 (2.2) 1 – 7	3.8 (2.2) 1 – 6	4.3 (2.1) 2 – 6	4.0 (0.8) 3 – 5	4.3 (1.5) 3 – 6	4.7 (2.3) 2 – 6

**Table 4H:** Four-week follow-up effect measures: Ayahuasca Preparation & Support Scale.

Ayahuasca Preparation & Support Scale: The first four questions are based on safety and support. The final two questions are based on preparation. The four-point scale answers were 1 = “not at all”; 2 = “a small amount”; 3 = “moderately”; 4 to “very much”. Items are averaged in each subscale.							
Outcome measure	Descriptive statistic	Product A (n=5)	Product B (n=4)	A/B (n=4)	Product C (low dose) (n=4)	Product C (high dose) (n=4, unless specified)	
		1 week post T*	1 week post T*	4 week post T*	1 week post T1	1 week post T2	4 week post T2 (n=3)
Safety and Support	Mean (SD) Min – Max	3.6 (0.2) 3.5 – 4.0	3.8 (0.2) 3.5 – 4.0	3.6 (0.4) 3.0 – 4.0	3.9 (0.1) 3.8 – 4.0	3.9 (0.3) 3.5 – 4.0	3.8 (0) 3.8 – 3.8
Preparation	Mean (SD) Min – Max	3.7 (0.4) 3.0 – 4.0	3.8 (0.5) 3.0 – 4.0	3.5 (0.6) 3.0 – 4.0	3.9 (0.3) 3.5 – 4.0	3.9 (0.3) 3.5 – 4.0	3.7 (0.6) 3.0 – 4.0

Base = baseline session

T = treatment session

T1 = treatment session 1

T2 = treatment session 2

T* = treatment session (either 1 or 2 depending on the random order of product A / product B)

Pre T = during the treatment session prior to the administration of the study medication

Post T = during the treatment session at the conclusion of the psychedelic experience (a minimum of 4 hours to 8 hours post dose)

SD = 1 standard deviation

Min = minimum

Max = maximum

N*=2, only 2 of the 4 participants completed the day 1 post treatment session 2 PANAS

## Discussion

### Interpretation

The primary objective of this study was to evaluate and compare three different formulations of a DMT/Harmala encapsulated product on a range of safety and efficacy parameters.

All three formulations demonstrated a good safety profile. Physical examination, vital signs monitoring, and pathology results did not yield findings of concern at any timepoint throughout the trial. Most AEs resolved within the treatment session or within 24 hours. There were 71 adverse events recorded, with most considered to be study medication-related. The most frequently occurring study medication-related AEs were adverse physical effects followed by adverse mental health effects. The adverse events reported in this study are consistent with other experimental studies of traditional Ayahuasca in healthy volunteers and clinical populations (59).

Traditional Ayahuasca formulations are known to induce nausea and vomiting, with estimates ranging between 60 – 96% of users (60, 61). Vomiting was infrequently recorded in our participant group (2 of 9 participants) which is a positive outcome from the perspective of suitability of a formulation for use in clinical contexts. In traditional use of ayahuasca, “purging” is considered an integral part of the therapeutic process (62). The reduced gastrointestinal effect observed in this study is potentially due to the oral preparation being provided in dried powder form via capsules, as opposed to the traditional liquid form. It remains to be seen if this lack of emetic action has an impact on the therapeutic potential of our Acacia based formulations in clinical populations.

The majority of mental health events occurred with one participant following ingestion of ACL-010 Formulation C (high dose). This participant experienced a number of emotional-cognitive adverse mental health effects (including transient suicidal ideation) during their psychedelic experience which resolved during the session after interventions administered by the therapists (breathing techniques, reassurance, physical holding and restraint, physical repositioning, and removing hazards to personal safety). Review of the participant’s file and notes from pre-treatment preparation sessions did not reveal any factors that could have predicted this participant’s challenging emotional and psychological experience. This suggests possible high variability in the inter-individual response to this formulation, however as our data is based on only four participants receiving ACL-010 Formulation C, drawing a definitive conclusion is difficult. Rossi et al. (63) discuss other cases where trial participants have had similarly intense and challenging experiences with Ayahuasca which were also resolved during the session without the need for pharmacological intervention. Although these cases are rare and the adverse events are transient, trial staff should be aware that some participants may be prone to these responses.

Reporting of AEs in clinical trials with psychedelics is in itself challenging because the framework for the reporting of AEs does not take account of the possibility that occurrences typically considered as AEs may be a part of the therapeutic process in this context. Separating these different types of events is difficult, as is consistent assessment, classification, and reporting (64). In our reporting we have used the standardized medical terminology for reporting AEs in clinical studies – Medical Dictionary for Regulatory Activities (MedDRA) (65), however we acknowledge that this is somewhat reductive. Extra notes to the AE table provide further clarification and context to some of the reported AEs.

Of particular interest to study investigators was the strength and quality of the psychedelic experience induced by the study formulations. A primary outcome measure of the study was the rating of each formulation on the MEQ which purports to measure the strength of a classic mystical experience (CME). Higher ratings of a mystical type experience have been found to be positively related to changes in well-being after a psychedelic experience. Average scores for both high and low dose ACL-010 Formulation C on the MEQ were higher than Formulations A and B on all subscales and within the range of a CME. In comparison to other studies reporting total MEQ scores associated with ayahuasca consumption, the formulation C high dose average score was marginally to significantly higher (53, 66, 67).

Responses on the Tolerability and Differential Experience scale indicate that strength of psychedelic experience of Formulations A and B were generally rated as weaker than previous experience with Ayahuasca whereas low dose ACL-010 Formulation C was rated as similar and high dose ACL-010 Formulation C as stronger. The subjective experience (quality) of all formulations was generally rated as similar to previous experience with Ayahuasca. Both high and low dose levels of ACL-010 Formulation C were rated as similar or more beneficial than previous experience with Ayahuasca, Formulations A tended to be rated as less beneficial than previous experience with Ayahuasca. Other acute effect measures (5D-ASC, and SIMO) indicated a stronger subjective effect associated with ACL-010 Formulation C (low and high dose).

Most four-week follow-up measures showed little difference between the 3 formulations (PANAS, K-10, DASS-21, ISI, TEPS, PIQ, IDS) and little change from baseline or week 1 post-dose values. It is possible that these effects measures are not particularly sensitive in non-clinical populations where baseline levels are quite low. However, scores for ACL-010 Formulation C (high) were considerably higher on a number of positive PEQ subscales (attitudes about life and self, mood, positive behavior, and spirituality) at both one week and 4 weeks post dose 2.

If DMT/Harmala formulations are to be used in clinical and /or research settings it is important to be able to quantify the dose of both substances prior to administration and consistently deliver the known dose over multiple time points. ACL-010 Formulation C used in this study was a highly purified and standardized formulation which allowed more precise quantification of the active ingredients in each capsule. The data tentatively indicates that this formulation delivered superior outcomes in terms of the strength of the psychedelic experience, which has been shown to be predictive of therapeutic effect (30, 31, 68, 69). The ability to produce an encapsulated product of high purity and consistency which can be readily titrated up or down as clinically indicated is a potentially beneficial consideration if the product is to be used in future clinical trials, and eventually in clinical contexts. Furthermore, the stability of traditional Ayahuasca beverages have been studied, and the harmala alkaloid component has been shown to degrade over time at a faster rate than the DMT component (70). Data from our stability studies indicate that these compounds when formulated in a pharmaceutical manner with appropriate excipients may potentially be more stable.

The therapist dyad, consisting of a psychiatrist and psychologist with extensive experience in psychedelic assisted psychotherapy, was a strength of our study, enhancing the value of the preparation and integration sessions, and the safety of trial participants during treatment sessions.

Our study protocol specified 2 treatment sessions with a minimum of 7 days between sessions. A washout period between 7–14 days has been used in a number of pharmacokinetic studies of DMT/harmine formulations (71–73) and given the half-life of the longest compound THH is approximately 6 hours there is no possible pharmacological carry-over effect. Nevertheless, it is possible that the subjective effects of the treatment may have cumulative effects. In fact, traditional ayahuasca ceremonies involve ingestion of the brew over multiple sessions. Treatment protocols for psychedelic assisted therapy are still emerging but typically involve one to three dosing sessions with the interval between sessions guided by both therapist and patient. Going forward, the optimal number of sessions and the interval between treatment sessions is likely to be determined by the mental health condition being treated and individual patient response to treatment.

A final comment is regarding the traditional use of these medicines. In the broader context of use of these compounds, it is important to consider culturally safe and effective treatment models. It is recommended that traditional custodians be ideally involved in protocol design through expert groups. Furthermore, regulatory bodies and sponsors should support this participation while addressing barriers such as cost to improve access and equity in clinical trials and treatments. See 74 for more discussion on this area.

### Limitations

This study has a number of limitations which include small sample size, lack of placebo, and the open label trial design. Study participants were healthy volunteers who were all mental health professionals with an interest in psychedelic assisted psychotherapy and previous experience with Ayahuasca. These factors limit the generalizability of results to the general population and clinical populations. Another possible confounder in our study is expectancy bias. One potential approach to minimize the impact of expectancy bias (from pre-existing positive beliefs inflating the treatment effect size) is via the use of the Stanford Expectancy Bias Scale (75) which can either exclude people pre-randomization with high positive treatment expectancy, or which can be used as a moderating covariate in efficacy analyses. Furthermore, it is noted that it is important to monitor the long-term effects of psychedelic administration via post-4-week follow-up assessments.

Our study was designed to test the safety, tolerability, physical, mental health and psychedelic effects of the three formulations in a naturalistic setting. Pharmacokinetics and pharmacodynamics were not assessed. The intrusive nature of procedures for frequent blood and urine sample collection were considered likely to detract from the psychotherapeutic nature and effects of the treatment sessions. A planned Phase 1 pharmacokinetic/pharmacodynamic study will be an integral part of ongoing formulation development prior to a Stage 2 trial.

## Conclusion

A unique aspect of the trial medication is that the DMT component of all formulations is derived from Acacia species. As far as the authors are aware, this is the first time a DMT/Harmala formulation derived from Acacia has been tested in a clinical trial. Our results are promising in terms of both the safety profile and subjective effects of the formulations, in particular ACL-010 Formulation C. Based on these preliminary findings, and considering of clinical trial data and the context of traditional dosage, we theories that ACL-010 Formulation C at a dose potentially midway between the low and high doses reported here could be most appropriate for further study. In summary, our results indicate that DMT formulations derived from the Acacia species represent a feasible alternative to the traditional Ayahuasca preparations (for reference, additional comparative data is currently in submission elsewhere). The caveat is the previously acknowledged small sample size, and therefore any conclusions regarding dosage, safety, and efficacy must be verified in an adequately powered randomized placebo-controlled trial.

## Data Availability

The data for this study are not available for sharing due to the small sample size creating an unacceptable risk of participant re-identification. Requests for access to summary data or aggregated results will be considered upon reasonable request. Requests to access the datasets should be directed to lisa.collins@svha.org.au.
