# Supplementary figures and images for: DMT and harmala alkaloids: an exploratory study of oral Acacia based formulations in healthy volunteers
Source: Front Psychiatry. 2025 Aug 15;16:1545915. doi: 10.3389/fpsyt.2025.1545915 (PMC12395344; doi:10.3389/fpsyt.2025.1545915)

Appendix 1: Schedule of Assessments and Procedures


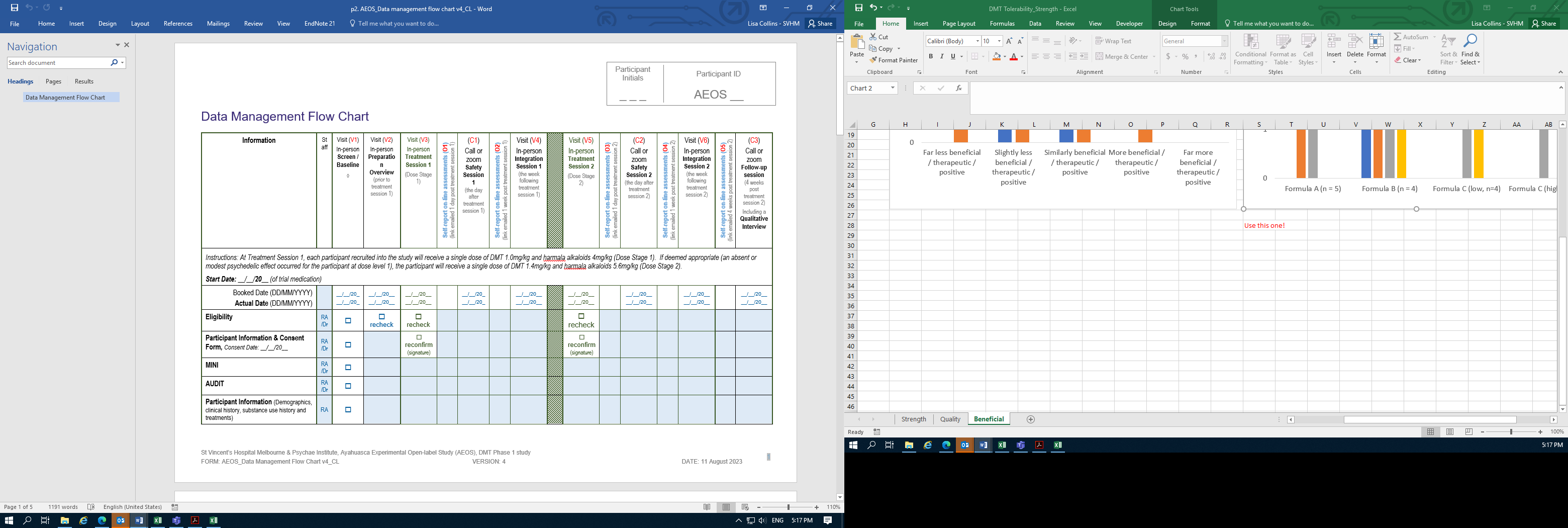


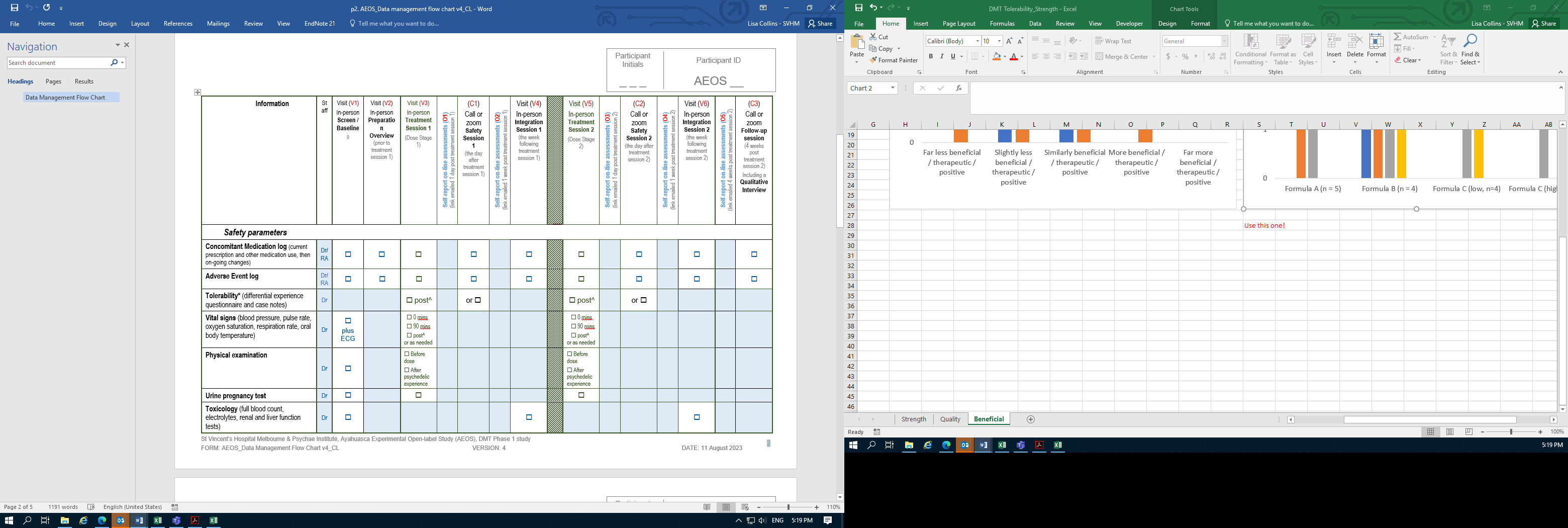


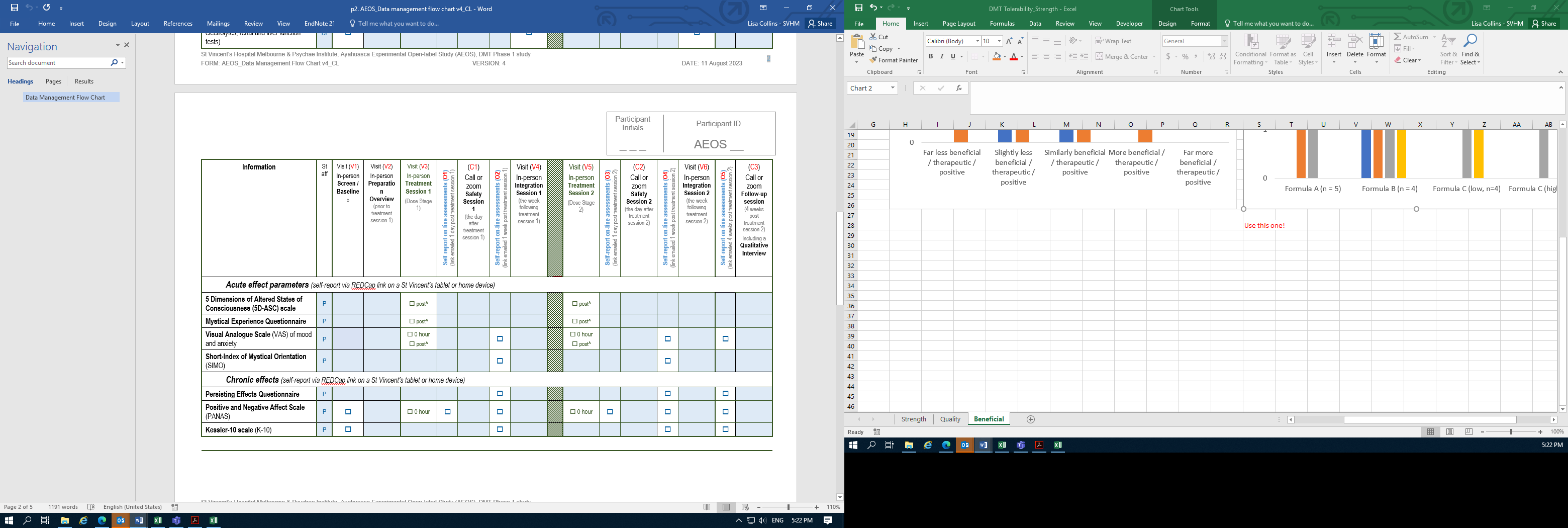


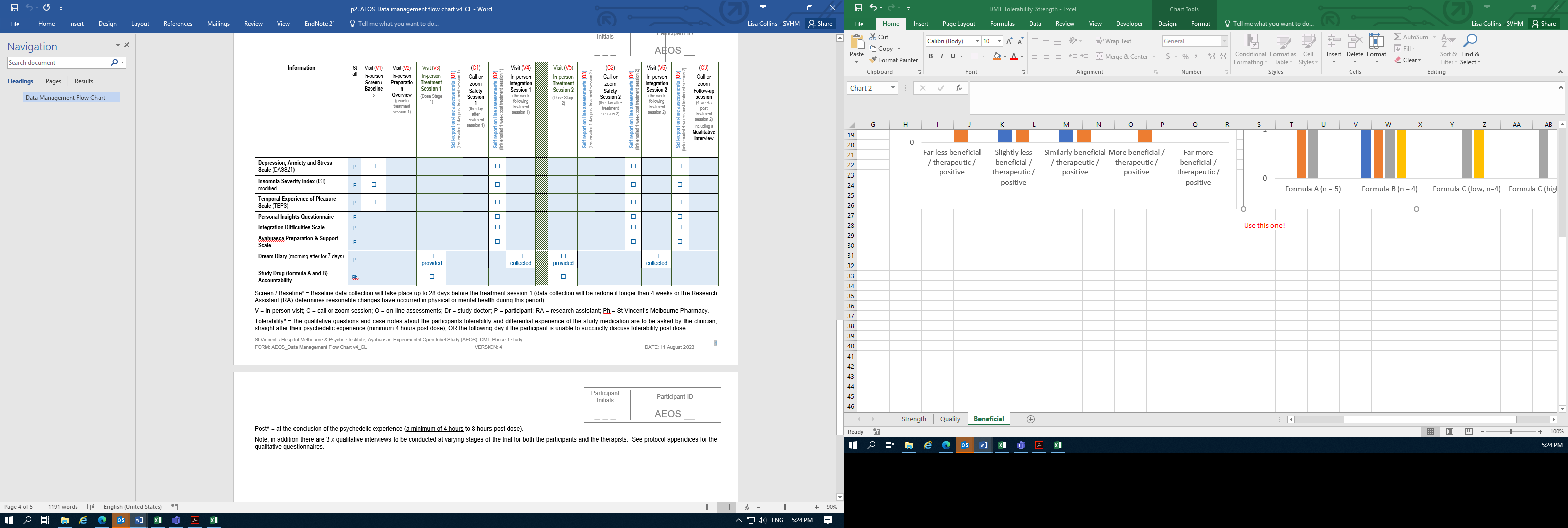


**
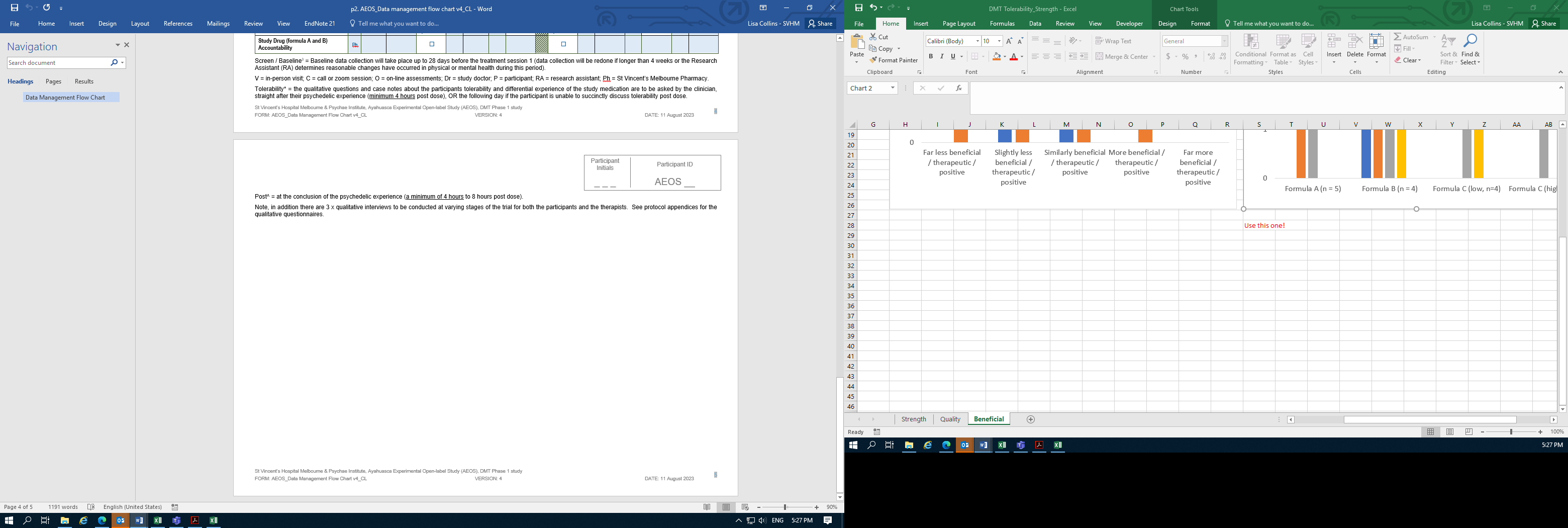
**

Supplement: Supplementary file 1 [file Table1.docx]

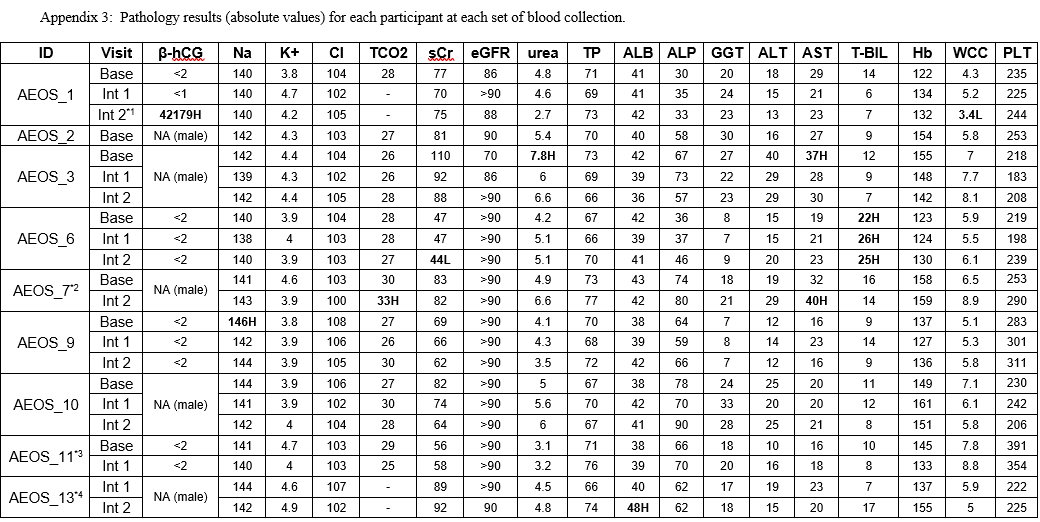

Supplement: Supplementary file 3 [file Table3.docx]
