# Supplementary material for: DMT and harmala alkaloids: an exploratory study of oral Acacia based formulations in healthy volunteers
Source: Front Psychiatry. 2025 Aug 15;16:1545915. doi: 10.3389/fpsyt.2025.1545915 (PMC12395344; doi:10.3389/fpsyt.2025.1545915)

Appendix 2: Vital signs tabulation (absolute values and change from baseline) at each set of observations. Plus average across participants for each formulation.

| **Vital Sign** | | **Baseline** (Day 0) | **Formulation A** | | | | | | **Formulation B** | | | | | |  | **Baseline** (Day 0) | **Formulation C (low)** | | | | **Formulation B (high)** | | | |
| --- | --- | --- | --- | --- | --- | --- | --- | --- | --- | --- | --- | --- | --- | --- | --- | --- | --- | --- | --- | --- | --- | --- | --- | --- |
|  |  |  | Preparatory | Pre-dose | Time 1 | Time 2 | Time 3 | Integration | Preparatory | Pre-dose | T1 | T2 | T3 | Integration |  |  | Pre-dose | T1 | T2 | T3 | Pre-dose | T1 | T2 | T3 |
| **Body Temperature (ºC)** | |  |  |  |  |  |  |  |  |  |  |  |  |  |  |  |  |  |  |  |  |  |  |  |
| Absolute values | AEOS_1 | 36.9 | 36.2 | 36.8 | 35.9 | 36.6 |  |  |  | 36.8 | 36.9 |  |  |  | AEOS_9 | 36.5 | 36.8 | 36.9 | 37.2 |  | 36.3 | 36.2 | 36.7 |  |
|  | AEOS_2 | 36.9 |  | 36.7 |  | 36.3 |  | 36.7 |  |  |  |  |  |  | AEOS_10 | 36.3 | 36.7 | 36.9 | 36.9 |  | 36.7 | 36.5 | 36.7 |  |
|  | AEOS_3 |  |  | 37.2 |  |  |  |  |  |  | 37 |  |  |  | AEOS_11 | 36.1 | 36.8 | 36.9 | 36.8 |  | 37.1 | 36.8 | 36.7 |  |
|  | AEOS_6 | 36.7 |  | 36.7 |  |  | 36.6 |  |  |  | 36.9 |  |  |  | AEOS_13 | 36.7 | 36.3 | - | 36.3 |  | 36.7 | 36.8 | 36.5 |  |
|  | AEOS_7 |  |  | 36.7 |  | 36.7 |  |  |  | 36.8 | 36.1 |  | 36.8 |  |  |  |  |  |  |  |  |  |  |  |
| Average |  | 36.8 | 36.2 | 36.8 | 35.9 | 36.5 | 36.6 | 36.7 |  | 36.9 | 36.5 |  | 36.7 |  |  | 36.4 | 36.7 | 36.9 | 36.8 |  | 36.7 | 36.6 | 36.7 |  |
| Std Dev |  | 0.12 | - | 0.22 | - | 0.21 | - | - |  | 0.22 | 0.57 |  | 0.14 |  |  | 0.26 | 0.24 | 0 | 0.37 |  | 0.33 | 0.29 | 0.1 |  |
| Changes from baseline | AEOS_1 |  | -0.7 | -0.1 | -1 | -0.3 |  |  |  | -0.1 | 0 |  |  |  | AEOS_9 |  | 0.3 | 0.4 | 0.7 |  | -0.2 | -0.3 | 0.2 |  |
|  | AEOS_2 |  |  | -0.2 |  | -0.6 |  | -0.2 |  |  |  |  |  |  | AEOS_10 |  | 0.4 | 0.6 | 0.6 |  | 0.4 | 0.2 | 0.4 |  |
|  | AEOS_3 |  |  |  |  |  |  |  |  |  |  |  |  |  | AEOS_11 |  | 0.7 | 0.8 | 0.7 |  | 1.0 | 0.7 | 0.6 |  |
|  | AEOS_6 |  |  | 0 |  |  |  |  |  |  | 0.2 |  |  |  | AEOS_13 |  | -0.4 |  | -0.4 |  | 0 | 0.1 | -0.2 |  |
|  | AEOS_7 |  |  |  |  |  |  |  |  |  |  |  |  |  |  |  |  |  |  |  |  |  |  |  |
| Average |  |  | -0.7 | -0.1 | -1 | -0.45 |  | -0.2 |  | -0.1 | 0.1 |  |  |  |  |  | 0.25 | 0.6 | 0.4 |  | 0.3 | 0.18 | 0.25 |  |
| Std Dev |  |  | - | 0.1 | - | 0.21 |  | - |  | - | 0.14 |  |  |  |  |  | 0.47 | 0.20 | 0.54 |  | 0.53 | 0.41 | 0.34 |  |
| **Heart rate**  **(beats / minute)** | |  |  |  |  |  |  |  |  |  |  |  |  |  |  |  |  |  |  |  |  |  |  |  |
| Absolute values | AEOS_1 | 52 | 51 | 76 | 55 | 69 |  |  |  | 65 | 62 |  |  |  | AEOS_9 | 70 | 77 | 63 | 80 |  | 83 | 69 | 90 |  |
|  | AEOS_2 | 77 | 56 | 72 | 59 | 47 | 58 | 77 |  |  |  |  |  |  | AEOS_10 | 57 | 57 | 55 | 67 |  | 48 | 45 | 76 |  |
|  | AEOS_3 | 65 |  | 69 | 59 | 61 |  |  |  | 69 | 62 | 61 |  |  | AEOS_11 | 71 | 78 | 85 | 63 |  | 72 | 79 | 66 |  |
|  | AEOS_6 | 79 | 64 | 72 | 76 | 68 | 87 |  |  | 100 | 68 | 88 |  |  | AEOS_13 | 74 | 72 | 82 | 79 |  | 81 | 72 | 88 |  |
|  | AEOS_7 | 51 |  | 75 | 73 | 89 |  |  |  | 76 | 60 | 78 | 78 |  |  |  |  |  |  |  |  |  |  |  |
| Average |  | 64.8 | 57 | 72.8 | 64.4 | 68.3 | 72.5 | 77 |  | 77.5 | 63.0 | 75.7 | 78.0 |  |  | 68 | 71 | 71.3 | 72.3 |  | 71 | 66.3 | 80 |  |
| Std Dev |  | 13.3 | 6.6 | 2.8 | 9.4 | 17.2 | 20.5 | - |  | 15.7 | 3.5 | 13.7 | - |  |  | 7.5 | 9.7 | 14.6 | 8.5 |  | 16.1 | 14.8 | 11.2 |  |
| Changes from baseline | AEOS_1 |  | -1 | 24 | 3 | 17 |  |  |  | 13 | 10 |  |  |  | AEOS_9 |  | 7 | -7 | 10 |  | 13 | -1 | 20 |  |
|  | AEOS_2 |  | -21 | -5 | -18 | -30 | -19 |  |  |  |  |  |  |  | AEOS_10 |  | 0 | -2 | 10 |  | -9 | -12 | 19 |  |
|  | AEOS_3 |  |  | 4 | -6 |  |  |  |  | 4 | -3 | -4 |  |  | AEOS_11 |  | 7 | 14 | -8 |  | 1 | 8 | -5 |  |
|  | AEOS_6 |  | -15 | -7 | -3 | -11 | 8 |  |  | 21 | -11 | 9 |  |  | AEOS_13 |  | -2 | 8 | 5 |  | 7 | -2 | 14 |  |
|  | AEOS_7 |  |  | 24 | 22 | 38 |  |  |  | 25 | 9 | 27 | 27 |  |  |  |  |  |  |  |  |  |  |  |
| Average |  |  | -12.3 | 8 | -0.4 | 3.5 | -5.5 |  |  | 15.8 | 1.3 | 10.7 | 27 |  |  |  | 3 | 3.3 | 4.3 |  | 3 | -1.8 | 12 |  |
| Std Dev |  |  | 10.3 | 15.2 | 14.7 | 30.0 | 19.1 |  |  | 9.3 | 10.1 | 15.6 | - |  |  |  | 4.7 | 9.5 | 8.5 |  | 9.4 | 8.2 | 11.6 |  |
| **Respiratory Rate (breaths / minute)** | |  |  |  |  |  |  |  |  |  |  |  |  |  |  |  |  |  |  |  |  |  |  |  |
| Absolute values | AEOS_1 | 16 | 13 | 14 | 12 | 13 |  |  |  | 16 | 14 |  |  |  | AEOS_9 | 16 | 16 | 14 | 16 |  | 16 | 15 | 16 |  |
|  | AEOS_2 | 16 |  | 16 |  |  |  | 16 |  |  |  |  |  |  | AEOS_10 | 16 | 16 | - | 18 |  | 16 | 17 | 14 |  |
|  | AEOS_3 |  |  | 16 | 14 |  |  |  |  | 16 |  |  |  |  | AEOS_11 | 16 | 16 | 16 | 16 |  | 16 | 22 | 18 |  |
|  | AEOS_6 | 12 | 14 | 16 | 13 | 16 | 16 |  |  | 16 | 14 | 13 |  |  | AEOS_13 | 16 | 16 | - | 16 |  | 16 | - | 18 |  |
|  | AEOS_7 | 16 |  | 16 | 12 | 16 |  |  |  | 16 | 12 | 14 | 13 |  |  |  |  |  |  |  |  |  |  |  |
| Average |  | 15 | 13.5 | 15.6 | 12.8 | 15 | 16 |  |  | 16 | 13.3 | 13.5 | 13 |  |  | 16 | 16 | 15 | 16.5 |  | 16 | 18 | 16.5 |  |
| Std Dev |  | 2 | 0.7 | 0.9 | 1.0 | 1.7 | - |  |  | 0 | 1.2 | 0.7 | - |  |  | 0 | 0 | 1.4 | 1 |  | 0 | 3.6 | 1.9 |  |
| Changes from baseline | AEOS_1 |  | -3 | -2 | -4 | -3 |  |  |  | 0 | -2 |  |  |  | AEOS_9 |  | 0 | -2 | 0 |  | 0 | -1 | 0 |  |
|  | AEOS_2 |  |  | 0 |  |  |  | 0 |  |  |  |  |  |  | AEOS_10 |  | 0 | - | 2 |  | 0 | 1 | -2 |  |
|  | AEOS_3 |  |  |  |  |  |  |  |  |  |  |  |  |  | AEOS_11 |  | 0 | 0 | 0 |  | 0 | 6 | 2 |  |
|  | AEOS_6 |  | 2 | 4 | 1 | 4 | 4 |  |  | 4 | 2 | 1 |  |  | AEOS_13 |  | 0 | - | 0 |  | 0 |  | 2 |  |
|  | AEOS_7 |  |  | 0 | -4 | 0 |  |  |  | 0 | -4 | -2 | -3 |  |  |  |  |  |  |  |  |  |  |  |
| Average |  |  | -0.5 | 0.5 | -2.3 | 0.3 | 4 | 0 |  | 1.3 | -1.3 | -0.5 | -3 |  |  |  | 0 | -1 | 0.5 |  | 0 | 2 | 0.5 |  |
| Std Dev |  |  | 3.5 | 2.5 | 2.9 | 3.5 | - | - |  | 2.3 | 3.1 | 2.1 | - |  |  |  | 0 | 1.4 | 1 |  | 0 | 3.6 | 1.9 |  |
| **Systolic Blood Pressure (mmHg)** | |  |  |  |  |  |  |  |  |  |  |  |  |  |  |  |  |  |  |  |  |  |  |  |
| Absolute values | AEOS_1 | 105 | 104 | 121 | 109 | 113 |  |  |  | 124 | 127 |  |  |  | AEOS_9 | 120 | 114 | 112 | 121 |  | 116 | 128 | 130 |  |
|  | AEOS_2 | 132 | 133 | 140 | 127 | 122 | 127 | 128 |  |  |  |  |  |  | AEOS_10 | 134 | 143 | 145 | 133 |  | 142 | 154 | 129 |  |
|  | AEOS_3 | 132 |  | 120 | 124 |  |  |  |  | 112 | 130 | 121 |  |  | AEOS_11 | 140 | 135 | 133 | 128 |  | 136 | 146 | 126 |  |
|  | AEOS_6 | 130 | 117 | 119 | 121 | 118 | 133 |  |  | 130 | 114 | 137 |  |  | AEOS_13 | 123 | 106 | 141 | 119 |  | 113 | 140 | 110 |  |
|  | AEOS_7 | 146 |  | 122 | 122 | 125 |  |  |  | 113 | 128 | 130 | 130 |  |  |  |  |  |  |  |  |  |  |  |
| Average |  | 129 | 118 | 124 | 120 | 119 | 130 | 128 |  | 120 | 125 | 129 | 130 |  |  | 129 | 124 | 132 | 125 |  | 127 | 142 | 124 |  |
| Std Dev |  | 14.9 | 14.5 | 8.8 | 6.9 | 5.2 | 4.2 | - |  | 8.7 | 7.3 | 8.0 | - |  |  | 9.4 | 17.4 | 14.7 | 6.4 |  | 14.4 | 11.0 | 9.3 |  |
| Changes from baseline | AEOS_1 | 105 | -1 | 16 | 4 | 8 |  |  |  | 19 | 22 |  |  |  | AEOS_9 |  | -6 | -8 | 1 |  | -4 | 8 | 10 |  |
|  | AEOS_2 | 132 | 1 | 8 | -5 | -10 | -5 | -4 |  |  |  |  |  |  | AEOS_10 |  | 9 | 11 | -1 |  | 8 | 20 | -5 |  |
|  | AEOS_3 | 132 |  | -12 | -8 |  |  |  |  | -20 | -2 | -11 |  |  | AEOS_11 |  | -5 | -7 | -12 |  | -4 | 6 | -14 |  |
|  | AEOS_6 | 130 | -13 | -11 | -9 | -12 | 3 |  |  | 0 | -16 | 7 |  |  | AEOS_13 |  | -17 | 18 | -4 |  | -10 | 17 | -13 |  |
|  | AEOS_7 | 146 |  | -24 | -24 | -21 |  |  |  | -33 | -18 | -16 | -16 |  |  |  |  |  |  |  |  |  |  |  |
| Average |  |  | -4.3 | -4.6 | -8.4 | -8.8 | -1 | -4 |  | -8.5 | -3.5 | -6.7 | -16 |  |  |  | -4.8 | 3.5 | -4 |  | -2.5 | 12.8 | -5.5 |  |
| Std Dev |  |  | 7.6 | 16.2 | 10.1 | 12.1 | 5.7 | 7 |  | 22.8 | 18.4 | 12.1 | - |  |  |  | 10.7 | 13.0 | 5.7 |  | 7.5 | 6.8 | 11.1 |  |
| **Diastolic Blood Pressure (mmHg)** | |  |  |  |  |  |  |  |  |  |  |  |  |  |  |  |  |  |  |  |  |  |  |  |
| Absolute values | AEOS_1 | 76 | 68 | 89 | 66 | 65 |  |  |  | 86 | 82 |  |  |  | AEOS_9 | 77 | 79 | 68 | 83 |  | 77 | 79 | 98 |  |
|  | AEOS_2 | 88 | 83 | 80 | 79 | 81 | 79 | 62 |  |  |  |  |  |  | AEOS_10 | 85 | 96 | 90 | 72 |  | 98 | 88 | 84 |  |
|  | AEOS_3 | 78 |  | 88 | 80 |  |  |  |  | 65 | 71 | 69 |  |  | AEOS_11 | 86 | 83 | 80 | 77 |  | 88 | 92 | 74 |  |
|  | AEOS_6 | 80 | 71 | 75 | 77 | 69 | 85 |  |  | 80 | 72 | 87 |  |  | AEOS_13 | 77 | 66 | 96 | 83 |  | 90 | 84 | 85 |  |
|  | AEOS_7 | 71 |  | 81 | 84 | 68 |  |  |  | 81 | 66 | 85 | 85 |  |  |  |  |  |  |  |  |  |  |  |
| Average |  | 79 | 74 | 83 | 77 | 71 | 82 | 62 |  | 78 | 73 | 80 | 85 |  |  | 81 | 81 | 84 | 79 |  | 88 | 86 | 85 |  |
| Std Dev |  | 6.2 | 7.9 | 5.9 | 6.8 | 7.0 | 4.2 | - |  | 9.1 | 6.7 | 9.9 | - |  |  | 4.9 | 12.4 | 12.3 | 5.3 |  | 8.7 | 5.6 | 9.8 |  |
| Changes from baseline | AEOS_1 |  | -8 | 13 | -10 | -11 |  |  |  | 10 | 6 |  |  |  | AEOS_9 |  | 2 | -9 | 6 |  | 0 | 2 | 21 |  |
|  | AEOS_2 |  | -5 | -8 | -9 | -7 | -9 | -26 |  |  |  |  |  |  | AEOS_10 |  | 11 | 5 | -13 |  | 13 | 3 | -1 |  |
|  | AEOS_3 |  |  | 10 | 2 |  |  |  |  | -13 | -7 | -9 |  |  | AEOS_11 |  | -3 | -6 | -9 |  | 2 | 6 | -12 |  |
|  | AEOS_6 |  | -9 | -5 | -3 | -11 | 5 |  |  | 0 | -8 | 7 |  |  | AEOS_13 |  | -11 | 19 | 6 |  | 13 | 7 | 8 |  |
|  | AEOS_7 |  |  | 10 | 13 | -3 |  |  |  | 10 | -5 | 14 | 14 |  |  |  |  |  |  |  |  |  |  |  |
| Average |  |  | -7.3 | 4 | -1.4 | -8 | -2 | -26 |  | 1.8 | -3.5 | 4 | 14 |  |  |  | -0.3 | 2.3 | -2.5 |  | 7 | 4.5 | 4 |  |
| Std Dev |  |  | 2.1 | 9.7 | 9.4 | 3.8 | 10.0 | - |  | 10.9 | 6.5 | 11.8 | - |  |  |  | 9.2 | 12.7 | 10.0 |  | 7.0 | 2.4 | 14.0 |  |
| **Oxygen Saturation (º)** | |  |  |  |  |  |  |  |  |  |  |  |  |  |  |  |  |  |  |  |  |  |  |  |
| Absolute values | AEOS_1 |  | 98 | 99 | 99 | 99 |  |  |  | 100 | 100 |  |  |  | AEOS_9 | 99 | 99 | 98 |  |  | 100 | 100 | 99 |  |
|  | AEOS_2 |  |  | 100 |  |  | 98 | 99 |  |  |  |  |  |  | AEOS_10 | 98 | 99 | 97 |  |  | 99 | 99 | 98 |  |
|  | AEOS_3 |  |  | 99 |  |  |  |  |  | 99 | 99 |  |  |  | AEOS_11 | 99 | 99 | 99 |  |  | 97 | 99 | 98 |  |
|  | AEOS_6 |  | 100 | 100 | 99 | 99 | 99 |  |  | 100 | 99 | 99 | 99 |  | AEOS_13 | 99 |  | 98 |  |  | 99 | 99 | 98 |  |
|  | AEOS_7 |  |  | 99 | 99 | 99 |  |  |  | 99 | 99 |  | 99 |  |  |  |  |  |  |  |  |  |  |  |
| Average |  |  |  |  |  |  |  |  |  |  |  |  |  |  |  |  |  |  |  |  |  |  |  |  |
| Std Dev |  |  |  |  |  |  |  |  |  |  |  |  |  |  |  |  |  |  |  |  |  |  |  |  |
| Changes from baseline | AEOS_1 |  |  | 1 | 1 | 1 |  |  |  | 2 | 2 |  |  |  | AEOS_9 |  | 0 | -1 |  |  | 1 | 1 | 0 |  |
|  | AEOS_2 |  |  |  |  |  |  |  |  |  |  |  |  |  | AEOS_10 |  | 1 | -1 |  |  | 1 | 1 | 0 |  |
|  | AEOS_3 |  |  |  |  |  |  |  |  |  |  |  |  |  | AEOS_11 |  | 0 | 0 |  |  | -2 | 0 | -1 |  |
|  | AEOS_6 |  |  | 0 | -1 | -1 | -1 |  |  | -1 |  |  |  |  | AEOS_13 |  |  | -1 |  |  | 0 | 0 | -1 |  |
|  | AEOS_7 |  |  |  |  |  |  |  |  |  |  |  |  |  |  |  |  |  |  |  |  |  |  |  |
| Average |  |  |  | 0.5 | 0 | 0 | -1 |  |  | 0.5 | 2 |  |  |  |  |  | 0.3 | -0.8 |  |  | 0 | 0.5 | -0.5 |  |
| Std Dev |  |  |  | 0.7 | 1.4 | 1.4 | - |  |  | 2.1 | - |  |  |  |  |  | 0.6 | 0.5 |  |  | 1.4 | 0.6 | 0.6 |  |


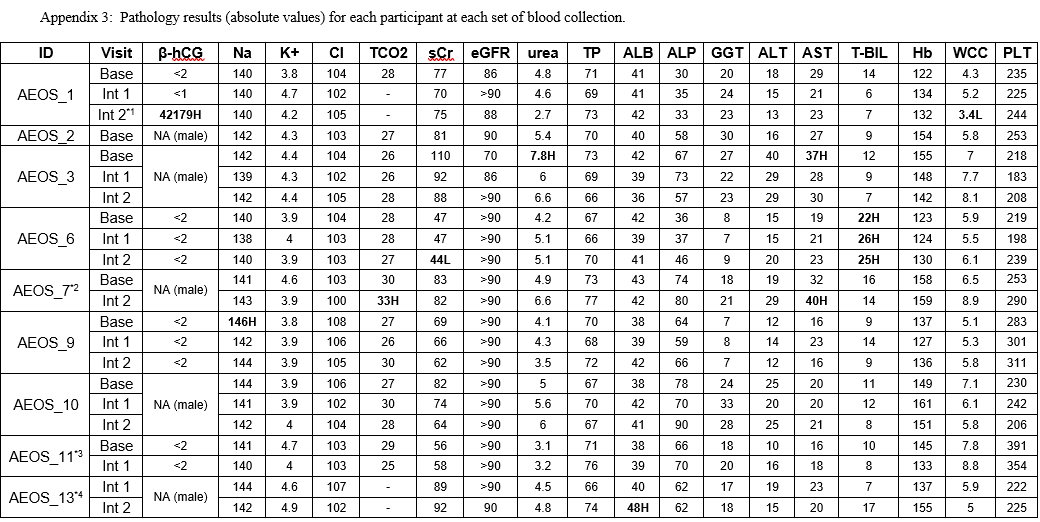

Supplement: Supplementary file 2 [file Table2.docx]
